# Transposase-DNA Complex Structures Reveal Mechanisms for Conjugative Transposition of Antibiotic Resistance

**DOI:** 10.1016/j.cell.2018.02.032

**Published:** 2018-03-22

**Authors:** Anna Rubio-Cosials, Eike C. Schulz, Lotte Lambertsen, Georgy Smyshlyaev, Carlos Rojas-Cordova, Kristoffer Forslund, Ezgi Karaca, Aleksandra Bebel, Peer Bork, Orsolya Barabas

**Affiliations:** 1 Structural and Computational Biology Unit, European Molecular Biology Laboratory (EMBL), 69117 Heidelberg, Germany; 2 Hamburg Outstation, European Molecular Biology Laboratory, 22603 Hamburg, Germany; 3 European Bioinformatics Institute (EMBL-EBI), European Molecular Biology Laboratory, Hinxton CB10 1SD, UK; 4 Izmir Biomedicine and Genome Center (IBG), 35340 Izmir, Turkey; 5 Max Delbrück Center for Molecular Medicine, 13125 Berlin, Germany; 6 Molecular Medicine Partnership Unit, 69120 Heidelberg, Germany; 7 Department of Bioinformatics, Biocenter, University of Würzburg, 97074 Würzburg, Germany

**Keywords:** DNA complex, crystallography, Tn1549 transposon, Tn916-like transposon family, conjugative transposition, tyrosine recombinase, antibiotic resistance, gene transfer, vancomycin, multidrug-resistant bacteria

## Abstract

Conjugative transposition drives the emergence of multidrug resistance in diverse bacterial pathogens, yet the mechanisms are poorly characterized. The Tn*1549* conjugative transposon propagates resistance to the antibiotic vancomycin used for severe drug-resistant infections. Here, we present four high-resolution structures of the conserved Y-transposase of Tn*1549* complexed with circular transposon DNA intermediates. The structures reveal individual transposition steps and explain how specific DNA distortion and cleavage mechanisms enable DNA strand exchange with an absolute minimum homology requirement. This appears to uniquely allow Tn*916*-like conjugative transposons to bypass DNA homology and insert into diverse genomic sites, expanding gene transfer. We further uncover a structural regulatory mechanism that prevents premature cleavage of the transposon DNA before a suitable target DNA is found and generate a peptide antagonist that interferes with the transposase-DNA structure to block transposition. Our results reveal mechanistic principles of conjugative transposition that could help control the spread of antibiotic resistance genes.

## Highlights

• Antibiotic resistance-carrying conjugative transposon integrase structure revealed
• DNA distortion and special cleavage site allow insertion into diverse genomic sites
• Key structural features are conserved among numerous conjugative transposons
• Structures uncover auto-inhibition, allowing transposition antagonist design

## Graphical Abstract

**Figure:**
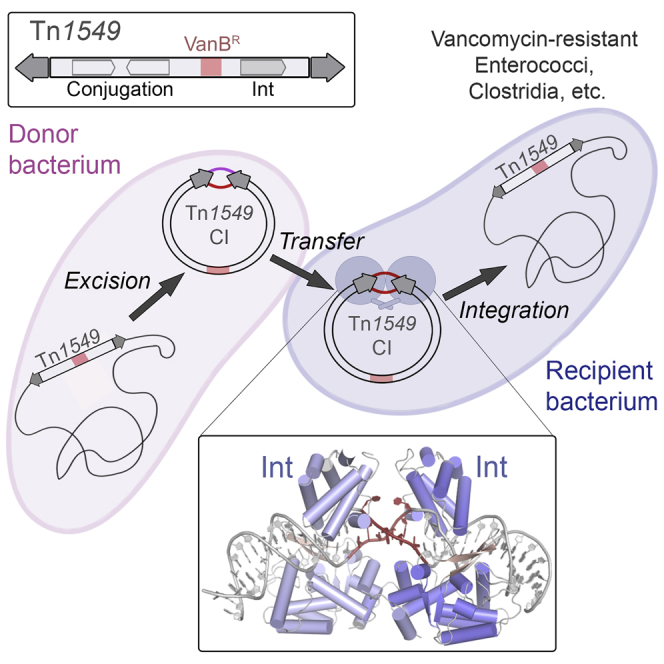

## Teaser

Structures of a conjugative transposase caught in the act reveal valuable mechanistic insight and point to potential strategies for limiting dissemination of antibiotic resistance.

## Introduction

DNA transposons are autonomous mobile genetic elements present in all kingdoms of life. In bacteria, transposons can carry antibiotic resistance genes and are major drivers of resistance spreading (van Hoek et al., 2011, Wozniak and Waldor, 2010). Transposition has been linked to the emergence of several multidrug-resistant opportunistic pathogens such as vancomycin-resistant *Enterococcus* (VRE), methicillin-resistant *Staphylococcus aureus* (MRSA), and extended spectrum β-lactamase-carrying *Enterobacteriaceae* (ESBL), which have become major health threats (Alekshun and Levy, 2007, Liu et al., 2016, Uemura et al., 2010).

Conjugative transposons (CTns; also referred to as integrative conjugative elements) constitute a major class of DNA transposons that can self-sufficiently move between bacterial genomes. They harbor resistance genes against many different antibiotics in diverse bacteria. Most CTns in Gram-positive pathogens belong to the large Tn*916*-like family, members of which confer resistance to all major antibiotic classes used against these bacteria (Bi et al., 2012, Roberts and Mullany, 2011). A prominent Tn*916*-like CTn is Tn*1549*, which is a major source of vancomycin resistance (VanB) in clinical VRE isolates (van Hal et al., 2016). As one of the leading causes of hospital-acquired infections, VRE causes diverse, difficult to treat infections (e.g., urinary infections, sepsis, and endocarditis), putting immunocompromised and transplant patients at critical risk (Agudelo Higuita and Huycke, 2014, Guzman Prieto et al., 2016). Transfer of vancomycin resistance via Tn*1549* has already been observed to various genera (Ballard et al., 2005, Launay et al., 2006), which is concerning as vancomycin is commonly used as a last resort antibiotic in the treatment of drug-resistant *Enterococcus*, *Clostridium*, and *Staphylococcus* infections (Rubinstein and Keynan, 2014).

**Figure 1:**
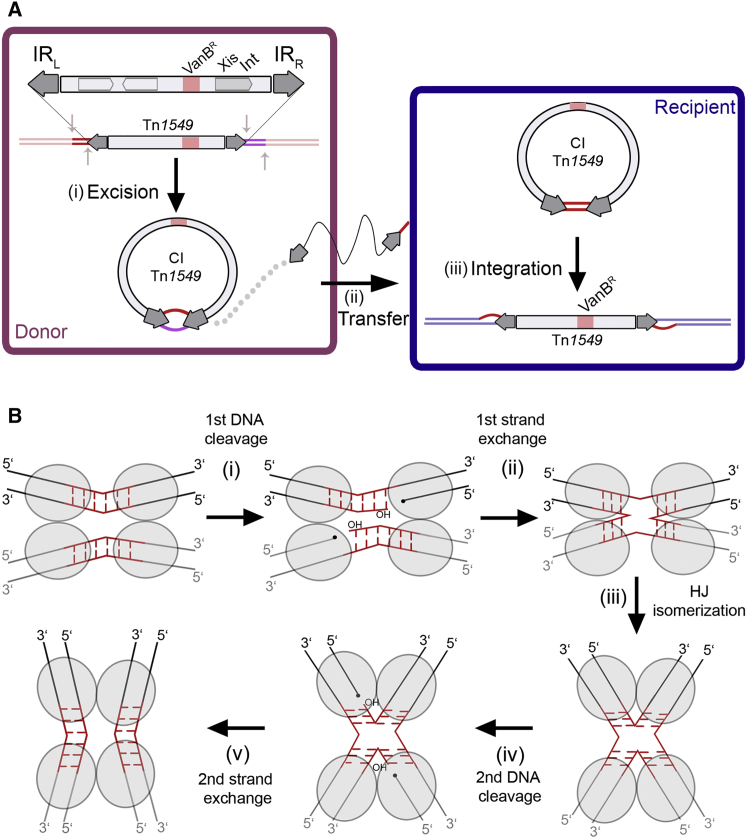
Tn*1549* Transposition and the Tyrosine Recombinase Chemistry (A) Schematics of Tn*1549* transposon movement. Tn*1549* encodes genes responsible for transposition (including Int), conjugation and mobilization (light gray boxes) and confers vancomycin resistance (VanB^R^, pink). Excision (i) in the donor cell (purple) creates the CI, with IR_L_ and IR_R_ (block arrows) joined by 5–7 nt heteroduplex; the donor DNA site is resealed. A single CI strand is transferred (ii) to a recipient bacterium (blue) by conjugation and replication re-creates the double-stranded CI, now with homoduplex at the crossover region. Integration (iii) in the recipient genome generates a new vancomycin resistant cell. (B) Tyrosine recombinases (gray ovals) recombine DNA substrates stepwise: (i) two proteins cleave one strand of each dsDNA using a tyrosine nucleophile, creating a covalent 3′-phosphotyrosyl bond (black dots) and a free 5′-hydroxyl (5′OH) group; (ii) the 5′ ends swap places and resolve the protein-DNA link on the partner strands, generating a four-way Holliday junction (HJ) intermediate; (iii) isomerization activates the second protein pair to cleavage (iv) and exchange (v) the other DNA strands, generating the final recombined products. Energetically inexpensive strand exchange requires homology in the substrates (red).

Despite their major role in drug resistance dissemination, CTns are among the least understood mobile DNA elements and gene transfer mechanisms. Microbiology and sequencing studies on two model CTns, Tn*916* and Tn*1549*, have suggested a general strategy for transposition involving excision from the host genome to create a circular transposon junction intermediate (CI), transfer of the CI to the recipient cell through conjugation, and finally integration in the recipient genome at random AT-rich locations (Tsvetkova et al., 2010, Wozniak and Waldor, 2010) (Figure 1A). However, due to limited biochemical and structural data on the molecular machinery involved in their movement, mechanistic details remain unknown. This has restricted our understanding of their behavior and our ability to limit their transfer to help tackle antibiotic resistance spreading.

The DNA cleavage and joining reactions required for CTn transposition are carried out by a transposase enzyme called integrase (Int). Most known CTns, including the Tn*916*-like family, have Y-transposases that use a conserved tyrosine residue to attack a phosphorous atom in the DNA backbone (Hickman and Dyda, 2015). Y-transposases belong to the tyrosine site-specific recombinase superfamily, members of which execute various biological functions including monomerization of phage, plasmid and chromosome multimers, resolution of hairpin telomeres, as well as mobilization of phages and transposons (Jayaram et al., 2015). Although most tyrosine recombinases use similar chemistry to recombine two specific DNA sites (Figure 1B) (Grindley et al., 2006), Tn*916*-like CTn enzymes have highly promiscuous DNA substrates, especially for integration, where they insert at random sites. How CTn transposases can overcome the low specificity of their substrates to achieve efficient transposition is currently unknown, although this is the key for spreading across an extremely diverse range of bacteria.

Here, we present the first crystal structures of a CTn transposase from Tn*1549* bound to DNA substrates. These reveal insights into CTn transposition and elucidate the molecular mechanisms used to recognize diverse transposon CIs and prepare them for integration at random genomic sites in diverse bacterial genomes. Our data also imply an auto-regulatory mechanism and make it possible to block Int activity *in vitro*, potentially opening new avenues to reduce the spread of antibiotic resistance.

## Results

### Structure of the Int-Circular Intermediate DNA Complex

**Figure 2:**
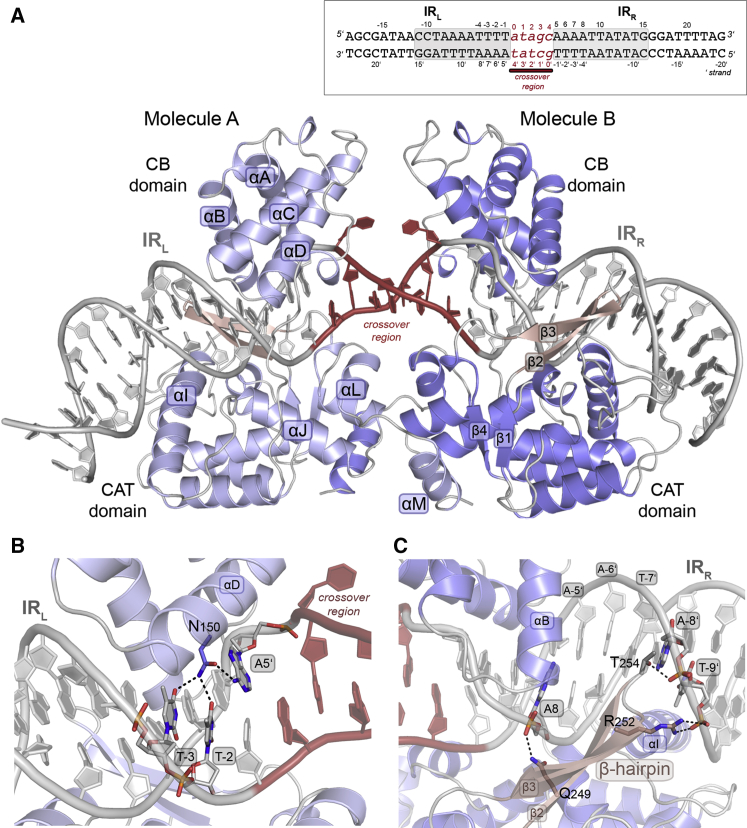
Transposon End Recognition by the Tn*1549* Int (A) Crystal structure of the R225K Int^82N^-CI5 DNA complex. Two Int molecules (A, B, two shades of blue) bind at IR_L_ and IR_R_ (gray), with the crossover region (red) at the center. Bases are numbered from the IR boundaries, numbers increase with the distance, negative numbers upstream, bottom strand marked with prime. CB and CAT are connected by a flexible linker. αA-D, αI, αJ, and the β-hairpin insertion (pink) interact with the DNA; αM is swapped between the two subunits. (B) Close-up of the base-specific DNA contacts of N150 with three terminal bases (sticks in atomic coloring). (C) Close-up of the β-hairpin. Q249, R252, and T254 (sticks) contact the DNA phosphate backbone. See also Figures S1 and S2 and Tables S1 and S2.

**Figure S1:**
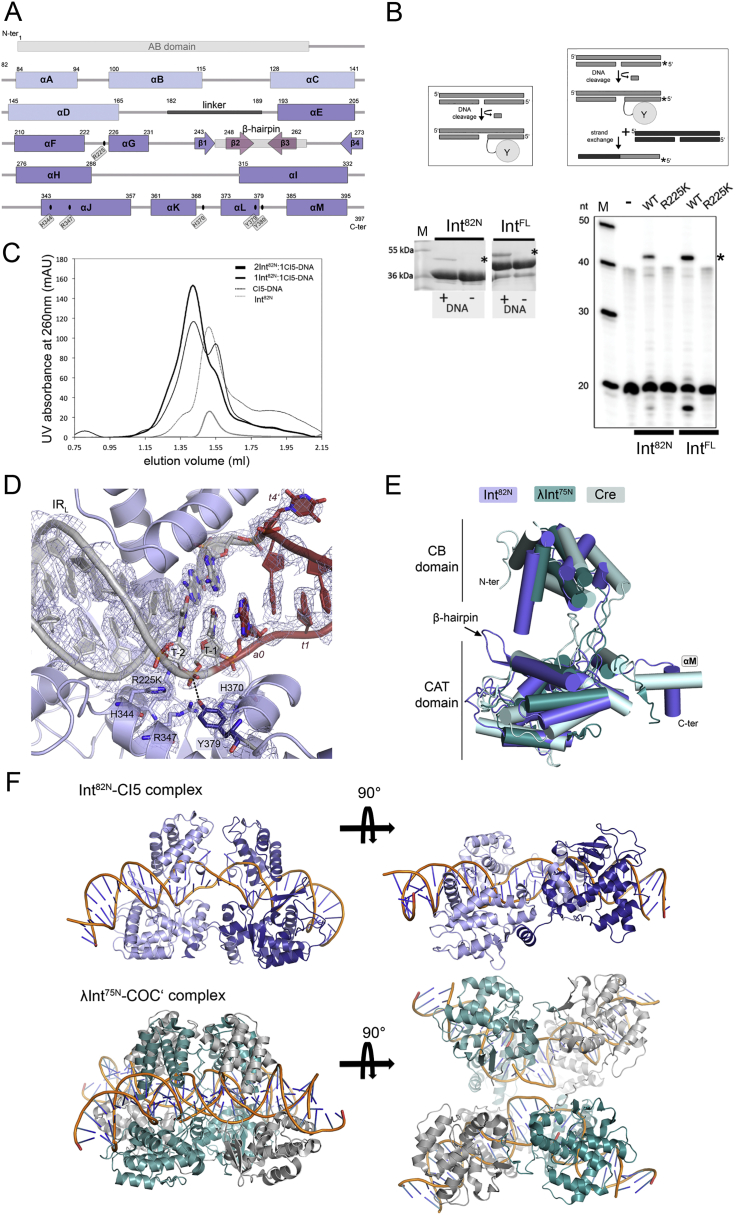
Reconstitution of the Int^82N^-CI5 Complex and Its Structural Features, Related to Figure 2 (A) Domain composition of Int. The N-terminal Arm-Binding domain (AB) (gray box) is followed by the Core Binding domain (CB) (αA-D, light blue boxes) and the Catalytic domain (CAT, consisting of αE-M and β1-4, dark blue boxes and arrows, respectively). The linker between CD and CAT is shown in black and the β-hairpin insertion is indicated as a gray box with dark purple arrows (β2-β3) inside. The catalytic residues are marked with black ovals and labeled. (B) *In vitro* reconstitution of Int activity. *Left:* DNA cleavage assays with Int^82N^ (aa 82-397) and Int^FL^ (aa 1-397) showing that both constructs cleave ‘suicide’ CI5 DNA. The substrates contain a nick in the DNA backbone downstream of the expected cleavage position (see schematics). Upon cleavage, 2 nucleotides diffuse away, trapping the covalent protein-DNA intermediate (gray oval attached to DNA) that can be resolved from unmodified Int on SDS-PAGE. Reactions with (+) or without (-) DNA are analyzed on a 12% SDS-PAGE gel. Relevant gel parts are cropped and shown next to each other. Asterisks indicate the band corresponding to the covalent protein-DNA product. *Right:* Results of DNA strand exchange assays with Int^82N^ and Int^FL^ show that both constructs create strand exchange products *in vitro*. Strand exchange reactions using 5′-^32^P radiolabeled (star) suicide CI5 DNA substrates (gray in the schematic) run on a denaturing 12% TBE-Urea PAGE gel. Following DNA cleavage, nucleophilic attack by the 5′OH of an unlabeled partner substrate (black) leads to a recombined product (black/gray bar). Ligation of the labeled substrate strand (20 nt) with unlabeled DNA results in a larger product (43 nt, marked with asterisk). The DNA band below the substrate corresponds to the cleavage product (18 nt). WT: wild-type protein, R225K: catalytic mutant, (-) no protein in the reaction. (C) Analytical size exclusion chromatography (SEC) of Int^82N^ with CI5 DNA at different protein:DNA ratios (on a Superdex 200 PC 3.2/30 column, GE Healthcare) reveals homogeneous complex at 2:1 ratio. Elution profiles for free CI DNA (peak at 1.50 mL), Int^82N^ alone (1.51 mL), complex at ratio 1 Int^82N^: 1 DNA (1.41 mL and 1.55 mL), and complex at ratio 2 Int^82N^: 1 DNA (1.41 mL) are compared. (D) Close-up of the active site in the Int^82N^-CI5 complex shows good quality experimental electron density. Simulated annealed composite omit map (2Fo-Fc, contoured at 1σ level) is shown for the catalytic residues (in sticks and atomic coloring) and DNA. (E) Structural superposition of Tn*1549* Int, Cre and λInt. One subunit is shown in cartoon representation (with helices shown as cylinders) for Int^82N^ (blue), λInt^75N^ (dark cyan, PDB: 1z19, aa 75-356) and Cre recombinase (light cyan, PDB: 1q3u). The overall fold is conserved among the three proteins, only the β-hairpin insertion is unique to Tn*1549* Int. (F) Comparison of the dimeric Int^82N^-CI5 complex (blue) with the tetrameric pre-cleavage λInt^75N^-COC’ complex (PDB: 1z19, cyan and gray). In the Int^82N^-CI5 complex, the C-terminal alpha helix is reciprocally exchanged between the two protein subunits, whereas the λInt^75N^-COC’ structure shows a cyclic exchange in homo-tetrameric assemblies.

To understand the structural basis of CTn transposition, we determined the crystal structure of the Tn*1549* Int in complex with a CI DNA substrate. Int contains 3 functional domains: an arm-binding domain (AB), a core DNA binding domain (CB), and a catalytic domain (CAT) (Figure S1A). CB and CAT are conserved in all tyrosine recombinases and are responsible for DNA binding at the Tn*1549* transposon ends, as well as for all DNA cleavage and ligation reactions during transposon excision and integration. The small and flexible AB domain is only present in a subset of tyrosine recombinases and is generally dispensable for DNA cleavage and strand exchange *in vitro* (Grindley et al., 2006, Sarkar et al., 2001). We confirmed that both the full-length Tn*1549* Int (Int^FL^) and an Int variant lacking the AB domain (aa 82–397; Int^82N^) catalyze CI DNA cleavage and strand exchange (Figure S1B). We obtained crystals of Int^82N^ bound to a 44 bp double-stranded DNA (dsDNA) oligonucleotide mimicking the CI (Figure S1C). The DNA (named CI5) represents a sequence from previous *in vivo* studies in *Clostridium* and contains the conserved left and right 11 bp long AT-rich imperfect inverted repeats (IR_L_ and IR_R_) that mark the ends of Tn*1549*, connected by a 5 bp homoduplex crossover region (*atagc*) (Domingo et al., 2005) (Figure 2A) as in the transposon integration substrate in the recipient cell (Figure 1A). To avoid heterogeneous DNA cleavage, we crystallized the catalytic R225K Int mutant.

The resulting 2.8 Å structure (Figures 2A and S1D; Table S1) revealed a dimer of Int^82N^ bound to one CI5 DNA. Each protein molecule covers one IR and the crossover region is located at the center between the two subunits. The CB and CAT domains form a clamp around the DNA and the two protein subunits face each other in a near-perfect 2-fold symmetric assembly. The dimer is held together by an extended intermolecular interface (1,409 Å^2^ with Δ^i^G = −30.2 kcal/mol; calculated by PISA; Krissinel and Henrick, 2007). The overall protein fold resembles other tyrosine recombinases, with two helical domains (Figures 2A and S1E). The C-terminal helix αM is swapped between the two subunits, interacting with a cleft on the surface of the partner (Figure 2A); αL that carries the nucleophile tyrosine interacts with its own subunit in *cis*. Similar arrangement was observed in the structures of Cre, XerH, and λ integrase (Bebel et al., 2016, Biswas et al., 2005, Guo et al., 1997) (Figures S1E and S1F) and was proposed to play a role in intersubunit communication. However, those structures showed a cyclic exchange in homo-tetrameric assemblies (Figure S1F), whereas in the Int^82N^-CI5 complex, the swap is reciprocal between the two protein subunits and constitutes a major interface holding the dimer together. The CI5 DNA molecule assumes a nearly straight conformation and the double helix is unwound and largely distorted at the crossover region (Figure 2A). This form is markedly different from the strongly bent DNA conformation seen in most structures of other tyrosine recombinases (Figure S1F).

### Transposon End Recognition

**Figure S2:**
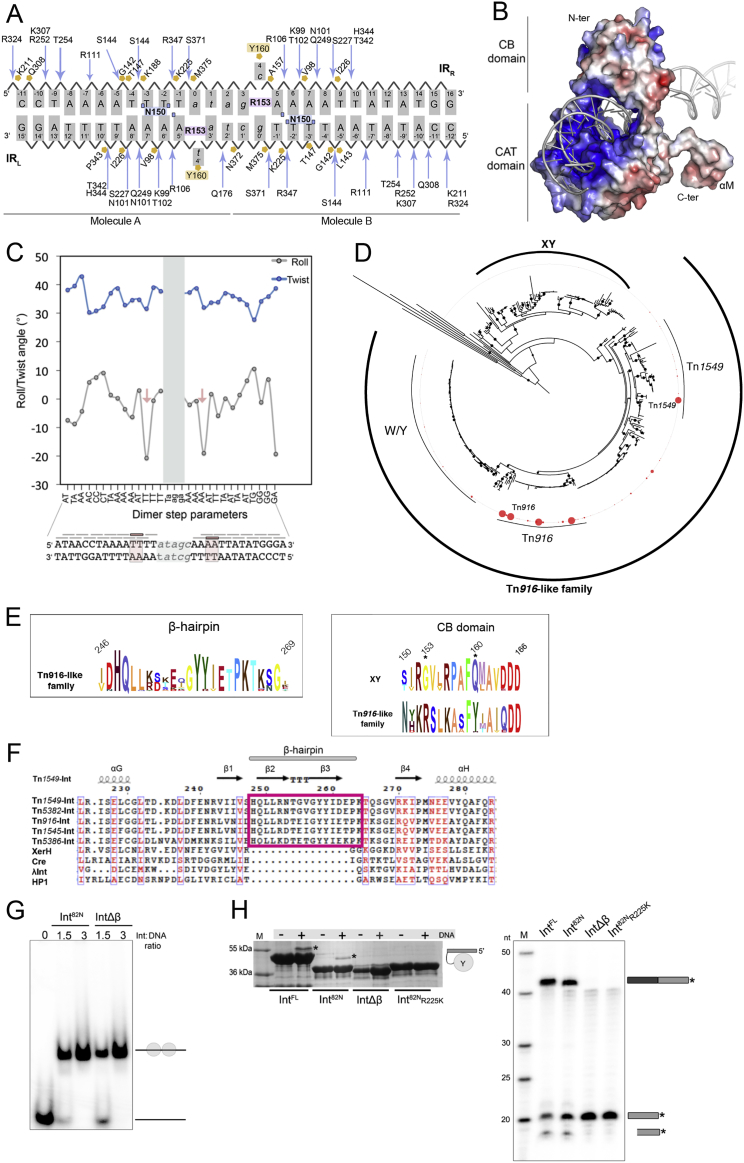
Structural Characteristics of Transposon End Binding, Related to Figure 2 (A) Schematic view of the protein-DNA contacts in the Int^82N^-CI5 crystal structure. The bases at the IRs are labeled in uppercase and the crossover region in lower case letters. IR_L_ is bound by Molecule A and IR_R_ by Molecule B (as indicated at the bottom). Blue arrows mark hydrogen bonding with the phosphate backbone and orange polygons indicate hydrophobic contacts. N150, the only residue performing base-specific contacts, is highlighted in blue with the interacting residues marked with blue squares. The residues involved in base flipping are shown in pink (R153) and orange (Y160). (B) Surface representation for the Int^82N^ subunit bound to IR_L_ DNA colored by electrostatic surface potential (blue, positive; red, negative; −5 to +5 kT/e isosurfaces) calculated using APBS in Pymol (Schrödinger). N- and C- termini are marked. Int^82N^ interacts extensively with DNA covering 8170 Å^2^ buried surface area at IR_L_ and 7810 Å^2^ at IR_R_ (calculated by PISA). (C) Graphical representation of roll (gray) and twist (blue) parameters for each local base pair step in CI5 DNA. Pink arrows in the plot indicate the last step in the A-tracts, with a marked decrease in the roll angle. The final A-s (A8’ and A8 in IR_L_ and IR_R_, respectively) have C3′ endo sugar pucker, a non-canonical backbone conformation in normal dsDNA that is frequently associated with DNA bending (Johnson et al., 2008). The crossover region (covered with a gray bar) was omitted from the calculation due to the flipped bases. The DNA sequence is shown under the plot, with pink boxes indicating the steps with a decreased roll angle. See also Table S2. (D) Phylogenetic tree of Tn*916*-like family transposases shows three different subgroups: Tn*1549*, Tn*916* (both with two consecutive tyrosines at the catalytic pocket, YY), and a third clade with the first tyrosine substituted by a tryptophan (called WY). XY marks a distinct related clade with only one conserved tyrosine. The red circles outside the tree indicate the number of distinct genomes in which the specific sequence is found. The largest circle marks sequences present in more than 100 genomes. Among these, Tn*916* and Tn*1549* Int-s are the most abundant, found in 3412 and 520 genomes, respectively. (E) Comparative sequence analysis reveals a high level of conservation for the β-hairpin (*left*) and for the residues involved in base flipping (R153 and Y160) among Tn*916*-like CTns, but not in the XY clade (*right*). N150, which recognizes the IR ends, is also highly conserved in the Tn*916*-like family. (F) Sequence alignment of Tn*916*-like Y-transposases and more distantly related tyrosine recombinases. The β-hairpin is present in Tn*916*-like Y-transposases, but absent in other tyrosine recombinases. The IntΔβ construct was created by replacing the β-hairpin insertion (H248-P263, pink frame) with a flexible two amino acid linker (GG). (G) Comparison of DNA binding by Int^82N^ and IntΔβ. Electrophoretic Mobility Shift Assay (EMSA) was performed with constant concentration of radiolabeled CI5 DNA (1 μM) and increasing concentrations of Int proteins (as indicated above the gel). Complexes are run on a native gel (TBE 4%–12% polyacrylamide gel). Schematics on the side mark the putative composition of each band. At 1.5 μM of protein, the amount of free DNA is higher for IntΔβ than for Int^82N^, indicating some decrease in DNA affinity upon deletion of the β-hairpin. (H) Results of *in vitro* cleavage (*left*) and strand exchange (*right*) assays with suicide CI5 DNA substrates demonstrating that the β-hairpin insertion is important for Int activity. See schematics in Figure S1B for the assay design. Cleavage assays are analyzed on SDS-PAGE, separating free Int and the covalent Int-DNA intermediate (asterisk). Strand exchange assays with radiolabeled suicide CI5 DNA substrates are analyzed on a denaturing TBE-Urea PAGE gel. Star denotes 5′-^32^P. Ligation of the labeled substrate strand (gray, 20 nt) with unlabeled DNA (black) results in a larger product (gray/black, 43 nt). The DNA band below the substrate corresponds to the cleavage product (18 nt). Int^FL^ and Int^82N^ readily catalyze CI DNA cleavage and strand exchange, but IntΔβ is compromised. Catalytic mutant R225K Int^82N^ is shown as negative control.

Both subunits of the Int^82N^ dimer interact extensively with DNA, each holding one transposon IR in the CI in a symmetric fashion. Despite several base pair differences in the sequence of IR_R_ and IR_L_, the protein-DNA interactions are nearly identical (Figure S2A). The CB domain inserts into the major groove at the inner parts of each IR, whereas CAT mostly interacts with the major groove at the outer parts (Figure 2). Together, the two domains encircle the DNA in a positively charged cleft, making numerous interactions with the DNA backbone (Figures S2A and S2B). Only one amino acid, N150 forms base-specific DNA contacts, creating a complex hydrogen-bonding network with the three terminal base pairs of the IRs (Figure 2B). These interactions help recognize the IR sequence near the cleavage sites, whereas the rest of the IRs form only backbone interactions.

The β2-β3 hairpin contributes a substantial part of the interactions with the IR DNA, mainly interacting with the DNA phosphate backbone at characteristic A-tracts in both IRs (Figure 2C). It is inserted between β1 and β4 in the loop region of the small β sheet of the canonical tyrosine recombinase fold, and is absent in the structures of previously characterized tyrosine recombinases (Figures S1A and S1E). The DNA conformation shows a slight curvature in both transposon ends, centered in the middle of each IR precisely at the end of the A-tracts (Figure S2C). This bend might be an intrinsic feature of the A-tracts that is recognized by the protein, or it may be induced by αI and the β-hairpin insertion. To explore the origin and importance of the β-hairpin, we performed bioinformatic analysis of CTn transposases and related tyrosine recombinases. As available transposon databases are incomplete, we first assembled a comprehensive set of Tn*916*-related transposases (350 non-redundant sequences with >40% similarity to Tn*1549* Int; Figure S2D). Comparative sequence analysis showed that the β-hairpin is present and highly conserved in Tn*916*-like family transposases, but absent in distantly related tyrosine recombinases (Figures S2E and S2F).

To test the role of the β-hairpin in Int function, we created an Int^82N^ variant (IntΔβ) by replacing the β-hairpin insertion with a short turn. IntΔβ retained DNA-binding (Figure S2G) but was compromised in CI DNA cleavage and strand exchange *in vitro* (Figure S2H). This shows that the β-hairpin is required for DNA recombination, likely by shaping the DNA substrates to promote Int activity.

### Int Melts the Center of the CI DNA

**Figure 3:**
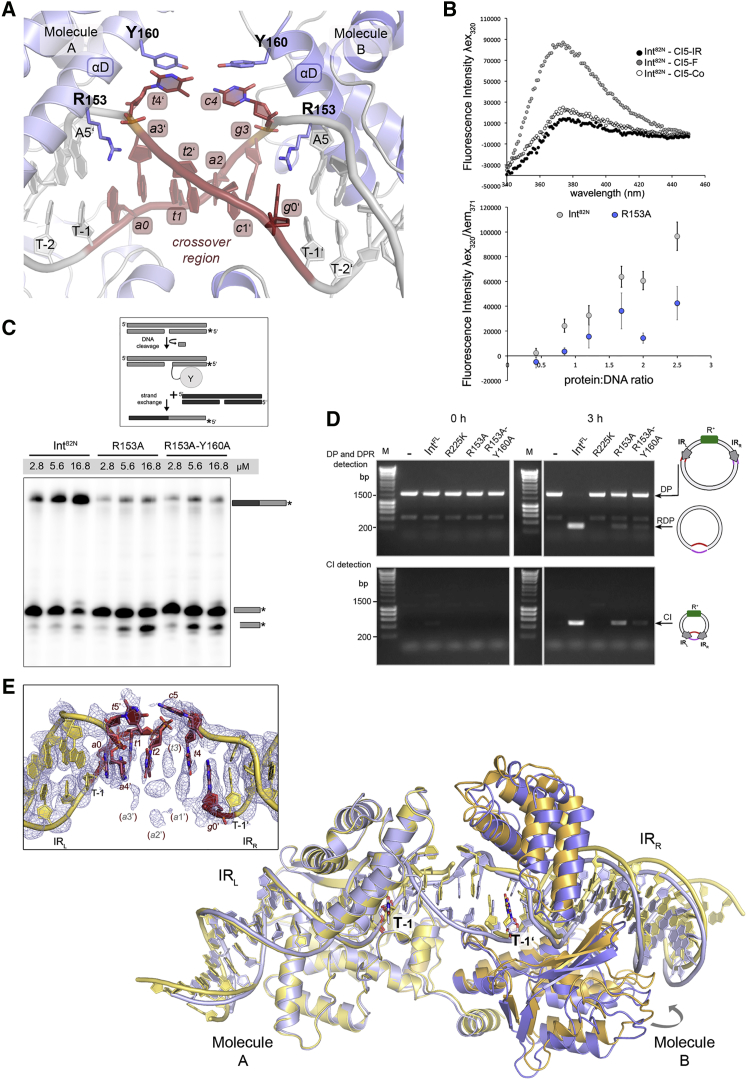
Int Triggers DNA Base Flipping, Unwinding, and Melting (A) Close-up of the crossover (red) in the Int^82N^-CI5 DNA complex. R153 invades the DNA after each IR, stacks with A5′/A5 and flips out *t*4′/*c*4 that are stabilized by Y160 (sticks). (B) 2AP fluorescence spectroscopy monitoring base flipping upon Int binding. Top: fluorescence emission spectra of Int^82N^-CI5 complexes (2:1 ratio) with 2AP in the IR (IR), in the crossover region (Co), and at the flipped base (F). Bottom: fluorescent signal of CI5-F with different amounts of Int^82N^ (gray) and R153A mutant (blue) at λem = 371 nm. Data are represented as mean ± SEM (Table S3). (C) Base flipping is required for efficient strand exchange. Results of strand exchange assays with Int^82N^ and base-flipping mutants (see also Table S4). Cleavage of the radiolabeled CI5 DNA (gray) and strand exchange with an unlabeled partner substrate (black) leads to a recombined product (gray/black) that is detected on denaturing PAGE. (D) Base flipping facilitates Tn*1549* excision *in vivo*. Excision of mini-Tn*1549* under Int^FL^ and Xis expression in *E. coli* is followed by PCR, monitoring formation of the CI and its loss from the donor plasmid (DP, RDP) (Lambertsen et al., 2018). Agarose gel showing PCR products from samples taken at 0 and 3 hr of Xis/Int^FL^ expression. R153A and R153A-Y160A mutants show a strong reduction of excision. Superfluous lanes were removed. Controls: R225K inactive mutant or no protein expressed (−). (E) Superposition of the R225K Int^82N^-CI6a (yellow) and Int^82N^-CI5 (blue) complex crystal structures (root-mean-square deviation [RMSD] for Cα atoms 0.54 Å) shows similar DNA distortions and a slight movement of Molecule B. The crossover DNA (red) has a high disorder in the electron density map of CI6a (composite omit map at 1σ) and some bases could not be located (inset; missing bases in brackets). See also Figures S3, S4, and Table S2.

**Figure S3:**
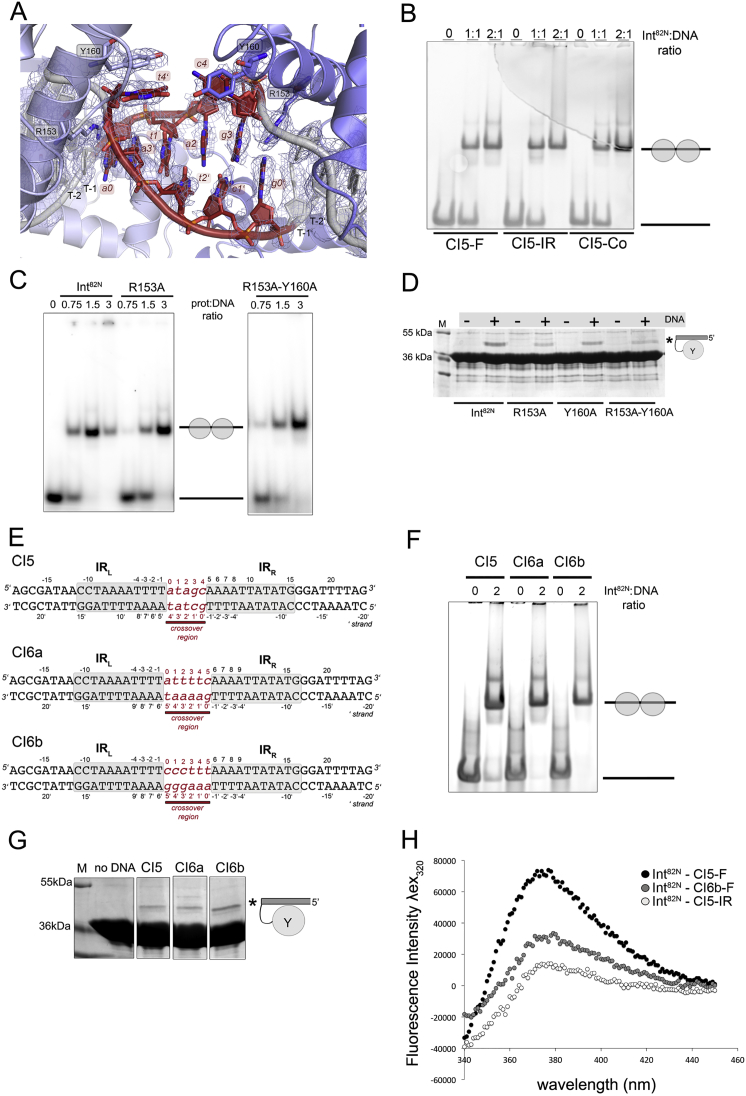
Int-Mediated Melting of the Crossover Region in Diverse CI DNA Substrates, Related to Figure 3 (A) Close-up of the crossover region (red) showing how DNA distortions destabilize base-pairing interactions. Several bases (*a*0-*a*3′-*t*1 and *g*3/*g*0’) form interdigitating base stacking instead of regular Watson-Crick base pairs. Simulated annealed composite omit map (2Fo-Fc, contoured at 1σ level) shows reduced quality electron density map at this region. (B) DNA binding assay with 2-aminopurine (2AP) modified CI5 DNA variants (CI5-F: 2AP at the flipped base, CI5-IR: 1nt inside IR and CI5-Co: 1nt inside the crossover region). Int^82N^ binds to all 2AP-modified DNA variants. (C) DNA binding assays with Int^82N^, R153A and R153A-Y160A mutants. Complexes with a constant concentration of radiolabeled CI5 DNA and different protein concentrations (0.75, 1.5 and 3 μM) are run on a native gel (TBE 4%–12% polyacrylamide gel). All mutants form complexes with DNA. (D) Int^82N^, R153A and R153A-Y160A mutants show similar DNA cleavage activity on suicide CI5 DNA. Gray circle attached to DNA marks the band (asterisk) corresponding to the covalent cleavage product on a 12% SDS-PAGE gel. See schematics in Figure S1B. (E) Tn*1549* CI sequences used in this study. IR_L_ and IR_R_ of Tn*1549* are highlighted in gray. Bases are numbered as described in Figure 2A. The crossover regions differ between the three sequences and are colored in red with lowercase letters. All CI sequences were derived from *in vivo* studies - CI5 from Domingo et al. (2005) and the CI6 sequences from Lambertsen et al., (2018). (F) Int^82N^ binding to different CI DNA sequences. Complexes show similar band shift, forming stable complexes with 2:1 protein:DNA ratio on a native gel (TBE 4%–12% PAGE gel). (G) Int^82N^ can cleave CI DNA independently of the crossover region sequence and length. DNA cleavage reactions with suicide CI DNA are analyzed on a 12% SDS-PAGE gel. Relevant gel segments are shown side by side. The covalent intermediate product is marked on the side. (H) Confirmation of base flipping in different CI DNA sequences. Fluorescence emission spectra of Int^82N^-CI5 DNA and Int^82N^-CI6b DNA complexes with 2AP modification at the flipped-out base are compared. y axis shows the corrected fluorescence intensity using an excitation wavelength (λex) of 320 nm. Complexes were prepared at 2 Int^82N^:1 DNA molar ratio. The fluorescence spectrum of the Int^82N^-CI5 complex with 2AP inside the IR is shown as control (see Figure 3B).

One of the most striking features of the Int^82N^-CI5 complex structure is the DNA conformation between the two IR sites. Here, the double helix is unwound and its backbone geometry and hydrogen-bonding pattern is largely distorted (Figures 3A and S3A). Distortion is initiated precisely at the boundary of the IR sequence, where R153 from the Int CB domain, invades the DNA and flips out the first base of the crossover region into an extra-helical position. From here, the distortion spans until the boundary of the other IR, where R153 from the other protein subunit flips out the first base of the crossover region on the other DNA strand. At both IRs, the R153 sidechain stacks with the terminal adenine base of the respective IR (A5′/A5 in IR_L_/IR_R_) and disrupts the continuity of base stacking. Base flipping also increases the DNA helical pitch (Table S2), resulting in unwinding of the central region. These distortions destabilize the base-pairing interactions and several bases form interdigitating base stacking instead of Watson-Crick base pairs. The electron density of the crossover DNA is less well defined, indicating that this part of the DNA is more flexible and partly disordered (Figure S3A).

Interestingly, the insertion of R153 and the resulting base flipping appear to be independent of the DNA sequence. In the two Int subunits, R153 inserts at different sequences on each side of the crossover region (Figure 3A), and the flipped-out bases are not recognized sequence specifically: they interact with Y160 in the CB domain via π-stacking (Figure 3A).

To confirm base flipping in solution, we performed fluorescence spectroscopy with DNA containing 2-aminopurine (2AP), a sensitive base-stacking sensor (Holz et al., 1998). When introducing 2AP at the flipped-out base (replacing *t*4′/*c*4; CI5-F), fluorescence markedly increased upon Int^82N^ addition (∼4.6-fold) (Figures 3B and S3B; Table S3). In turn, with 2AP inside the IR (at A5′/A5; CI5-IR) or in the crossover region (*a*3′/*g*3; CI5-Co), Int^82N^ had little effect. Thus, 2AP at the crossover boundary is specifically flipped into an unstacked extra-helical position upon Int binding. Consistent with the critical role of Int’s R153 in base flipping, substituting this residue with alanine drastically reduced fluorescence (∼5-fold; Figure 3B and Table S3).

### Efficient Transposition Requires Base Flipping *In Vitro* and *In Vivo*

To test the functional significance of base flipping on Int activity, we mutated R153 alone or together with Y160. Alanine substitutions only moderately reduced DNA binding and cleavage compared to unmutated Int^82N^ (Figures S3C and S3D). However, both mutants showed a severe decrease in strand exchange activity *in vitro* (Figure 3C; Table S4), suggesting that base flipping is particularly important for strand exchange. To test the effect of base flipping on transposition *in vivo*, we assayed excision of a mini-Tn*1549* transposon (Lambertsen et al., 2018) with R153A and R153A-Y160A Int^FL^ mutants in *E. coli*. Both mutants were highly compromised in excision, with very little CI generated and most transposon substrate left unprocessed (Figure 3D). This is consistent with the structural roles of R153 and Y160 in the Int-CI complex and shows that transposase-mediated base flipping and duplex melting play an important role during Tn*1549* transposition. Sequence alignments of Tn*916*-like CTns also revealed a strong conservation of R153 and Y160 across the family (Figure S2E).

### Int Dimers Accommodate CI DNA with Different Crossover Sequences and Length

During Tn*1549* transposition, excision generates CIs with different crossover sequences and lengths (5–7 nt) that can all be readily integrated at new genomic locations (Domingo et al., 2005, Launay et al., 2006). To understand how Int can accommodate different CIs, we determined the structure of two additional Int^82N^-CI complexes. DNA sequences contained IR_L_ and IR_R_ sites connected by 6 bp homoduplex crossover regions of different sequence (CI6a and CI6b, Figure S3E) (Lambertsen et al., 2018). Int^82N^ bound all CIs with similar affinity and stoichiometry (Figure S3F) and cleaved them *in vitro*, irrespective of their crossover sequence and length (Figure S3G).

**Figure S4:**
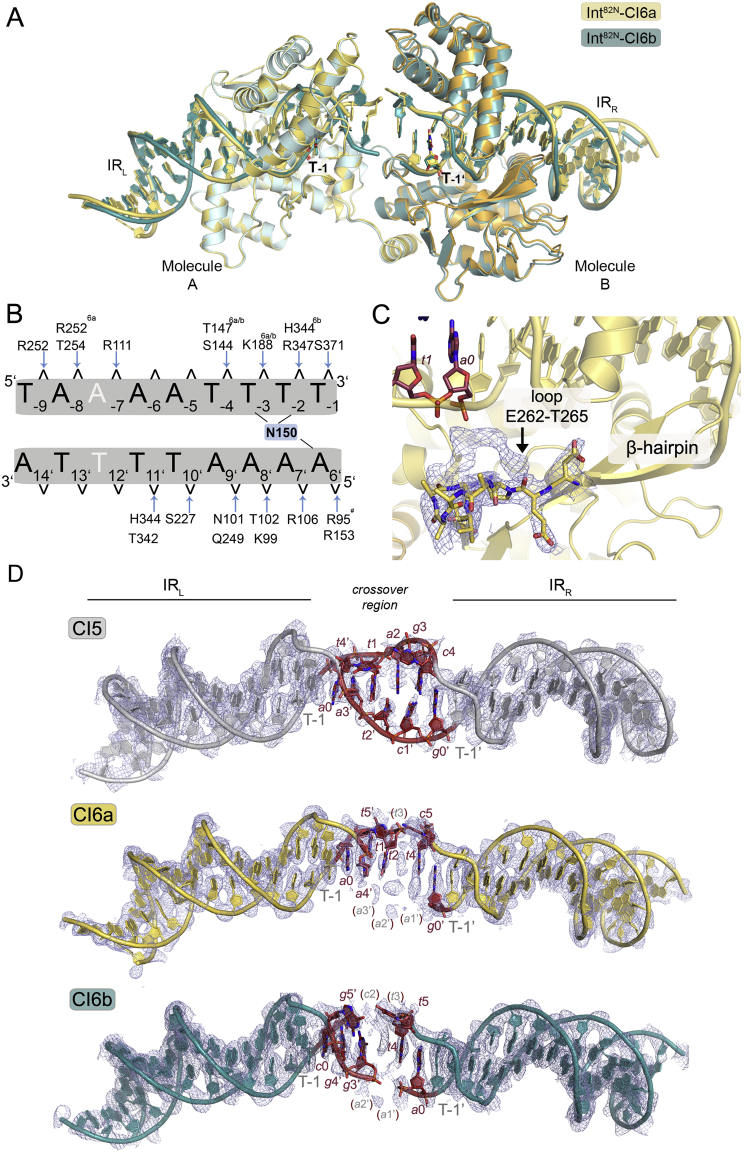
Structural Insights into the Recognition of Different Crossover Sequences and Lengths in Diverse Transposon CIs, Related to Figure 3 (A) Superposition of the Int^82N^-CI6b complex (cyan) and the Int^82N^-CI6a complex (yellow), with Molecule A as a reference, shows high similarity (RMSD 0.34 Å for Cα). Both crossover regions show high disorder and the central bases could not be built. The DNA appears to form interdigitating base stacking as in the Int^82N^-CI5 complex, although its path could not be fully resolved. (B) Comparative schematic representation of the Int^82N^-DNA contacts in the CI5, CI6a and CI6b structures. The DNA recognition pattern is conserved independent of the sequence and length of the crossover region. The CI6 IR_L_ sequence is shown. IR_R_ differs in the base highlighted in white. Blue arrows indicate hydrogen bonding with the phosphate backbone (contact cut-off 3.5Å). Base-specific DNA contacts of N150 are highlighted in blue with the interacting bases marked. Interactions that are present in some but not all complexes are marked with superscript: The interaction with R252 is shifted one nucleotide downstream in the CI6a structure; T147 forms hydrophobic contacts in CI5; N101 makes a water-mediated contact in CI6a; K188 contacts the T-3 phosphate in IR_R_ in CI6a and in both IRs in CI6b; R95 contacts (#) are absent at IR_R_. (C) Close-up of the β-hairpin insertion shows two alternative conformations for the final small loop (E262-T265, sticks) in the electron density maps in the CI6 structures (shown for CI6a). Simulated annealed composite omit map (2Fo-Fc, contoured at 1σ level) is shown for the loop. (D) Simulated annealed composite omit electron density maps reveal higher disorder in the CI6 structures. The map is shown at 1σ level, together with a cartoon representation for the three CI DNA molecules: CI5 (gray), CI6a (yellow) and CI6b (cyan). Bases at the crossover region are shown in atomic representation in red. Missing bases are indicated with gray letters in red brackets.

The two Int^82N^-CI6 structures were solved at 2.7 Å resolution (Figures 3E and S4A; Table S1) and show a similar architecture to the CI5 complex with two protein subunits binding one CI DNA. The structure of the protein molecules and their interactions with the DNA are practically identical (Figure S4B). One difference is in the small loop after the β-hairpin, which shows an additional conformation in the CI6 structures located near the DNA crossover region (Figure S4C). Another difference is the relative positioning of the two protein subunits in the dimer. In the Int^82N^-CI6 complexes, one subunit is shifted with the CAT domains ∼2–3 Å further apart than in the Int^82N^-CI5 complex (Figure 3E), reflecting small flexibility within the dimer. This shift is much less than expected to be imposed by the additional base in CI6 (∼6 Å shift and ∼35° “helical phase” change between the two Int binding sites in B-form DNA) implying that changes in the protein arrangement are not sufficient to accommodate the variations in the crossover DNA and changes in the DNA conformation are also required.

In both Int^82N^-CI6 complexes, the DNA assumes a similar conformation as in the CI5 complex. Due to unwinding of the crossover region, Int maintains the same interactions in the two IR sites irrespective of the length of the crossover region. R153 invades the DNA double helix, flips out the first base of the crossover region and disrupts base stacking and pairing. Even though the flipped-out bases are different in the CI6b structure (*g*/*t* instead of *t*/*c*), they form very similar interactions and fluorescence spectroscopy with 2AP-substituted CI6b DNA probes confirmed base flipping in solution (Figure S3H). The maps at the crossover region in the CI6 structures are particularly poorly defined (Figure S4D), suggesting that flexibility in this region helps accommodate CIs with different crossover lengths. Our two CI6 structures also show that different CI sequences can be accommodated in the center of the Int dimer similarly, again due to the distortions and flexibility of the crossover region.

### Active Site Architecture

**Figure 4:**
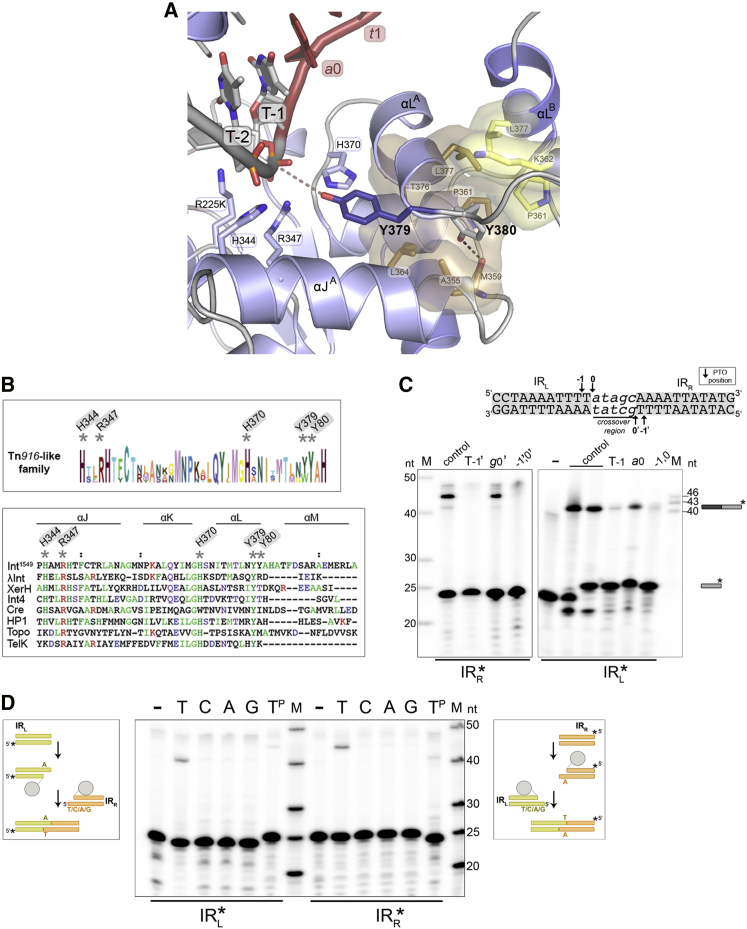
Int Cleaves inside the Conserved IRs (A) Close-up of the R225K Int^82N^-CI5 active site. All catalytic residues (sticks) assemble around the phosphate group of T-1. Y379 (dark blue) points toward the phosphate (4.9–5.4 Å distance, dashes); Y380 (gray) is anchored in a conserved hydrophobic pocket on the subunit interface, hydrogen bonding with M359 at the base of this pocket (black dashes) and with L377 and K362 in the backbone. (B) Sequence LOGO of Tn*916*-like CTn transposases (top; see Figure S2D for phylogenetic tree) shows that both tyrosines are conserved, but Y379 can be replaced with a tryptophan (W). Canonical tyrosine recombinases only have one tyrosine as seen in the structural alignment (bottom). (C) PTO mapping at IR_R_ (left) or IR_L_ (right) in ligation or strand exchange assays, respectively. PTO at T-1/T-1′ inside the IRs blocks DNA cleavage and strand exchange by Int^82N^. Control: no PTO; (−): no protein. (D) Strand ligation assays show that a terminal thymine at the incoming 5′OH is needed for strand exchange. 5′-radiolabeled IR_L_ (yellow, left) or IR_R_ (orange, right) half-site substrate is cleaved and ligated to an unlabeled partner substrate. Reactions with Int^82N^ and partner substrates with different base at the 5′OH (T/C/A/G) analyzed on denaturing PAGE. T^P^: 5′-phosphorylated DNA; (−) no protein. See also Figure S5.

**Figure S5:**
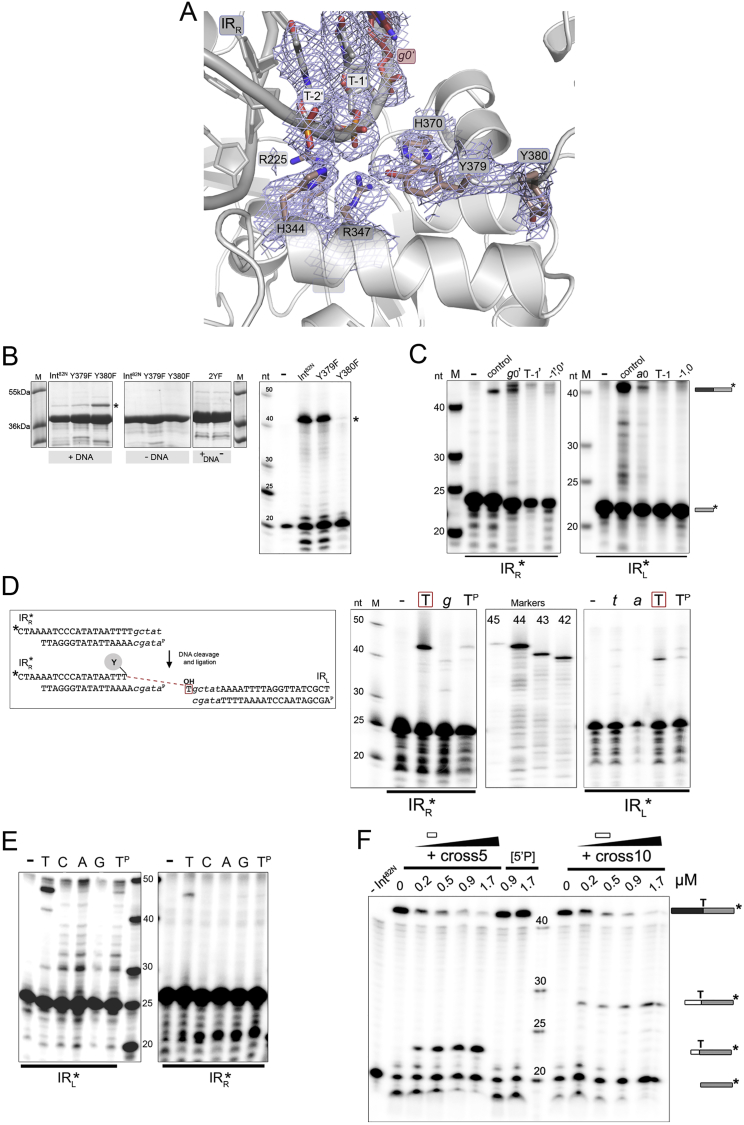
Characterization of the Int Active Site and Its DNA Cleavage and Strand Exchange Specificity, Related to Figure 4 (A) Close-up of the active site in the wild-type Int^82N^-CI5 complex structure shows the same architecture as in the R225K mutant. Simulated annealed 2Fo-Fc composite omit map contoured at 1σ level. As before, Y379 points toward the DNA at the phosphate of T-1/T-1’ (at 5.2-5.8 Å distance). R225 forms a hydrogen bond with the scissile phosphate (T-1/T-1’). The structures superpose with RMSD of 0.5020 Å for Cα atoms in Molecule A. (B) *In vitro* activity assays with tyrosine mutants Y379F and Y380F, and the double mutant 2YF with both tyrosines mutated to phenylalanine. DNA cleavage reactions (*left*) with suicide CI5 DNA are analyzed on 12% SDS-PAGE gel. Asterisk marks the covalent cleavage product. Both Y379F and Y380F mutants cleave DNA, whereas the double mutant is inactive. Strand exchange assays (*right*) with suicide CI5 DNA are run on denaturing 12% TBE-Urea gel. Irrelevant gel segments were eliminated. The recombination product is indicated with an asterisk. Here, the two single mutants show different behavior. (-): control lane without protein in the reaction. (C) Phosphorothioate (PTO) modification at position -1 inside the transposon blocks DNA cleavage and strand exchange in CI6a DNA. PTO modification is introduced at T-1/T-1’, at the neighboring position *a*0/*g*0’, or both (-1’,0’ and -1,0) (as for CI5 DNA in Figure 4C). Reactions are run on denaturing 12% TBE-Urea gel. Substrates and products are marked with schematics as in Figures S2H and 4C. (-): reaction without protein; control: reaction without PTO modification. (D) Ligation assays with truncated partner DNA confirm the need for cleavage 5′ of T-1/T-1’. Schematics show the experimental design with IR_R_ labeled. Two unlabeled IR_L_ partner substrates were tested: one with T-1’ and the other without (marked with red box). Reactions are run on denaturing 12% TBE-Urea PAGE gel with radiolabeled IR_R_ (on the *left*) and IR_L_ (on the *right*). Products are only observed with T-1/T-1’ present. (-) reactions without protein. T^P^ indicates reactions with the 5′ phosphorylated partner substrates. (E) Thymine at the 5’ is also necessary for strand exchange with CI6a DNA. Ligation reactions are visualized on denaturing 12% TBE-Urea PAGE gel. Reactions with radiolabeled IR_L_ (*left*) and IR_R_ (*right*) are shown. (F) A single stranded DNA (ssDNA) competes with Int-mediated recombination. Int^82N^ at 40 μM concentration was incubated with 0.5 μM CI5 DNA and increasing concentrations (0.2-1.7 μM, as marked above the gel) of unlabeled ssDNA that mimic the crossover region sequence (5nt length for cross5 and 10nt for cross10, both ending with the essential thymine at the 5′). The CI5 DNA substrate has a radiolabeled 20nt strand (short gray bar), that upon ligation with its partner substrate creates a 43nt recombination product (black/gray bar). Addition of cross5 or cross10 ssDNA blocks correct strand exchange and results in alternative products of 23 nt or 28 nt, respectively (white/gray bar). Controls: 5′P, 5′-phosphorylated DNA; -Int^82N^, no protein in the reaction.

In the active site of each Int subunit, several conserved residues are assembled around the DNA scissile phosphate, together forming a catalytic pocket characteristic of tyrosine recombinases. These include the catalytic triad K225 (natively R), H344, and R347, as well as H370 (Figures 4A and S1D). The active site architecture is very similar in the two protein subunits and in all three structures. We also determined the structure of the wild-type Int^82N^-CI5 complex (2.8 Å), which shows practically identical arrangement except for R225 that here contacts the scissile phosphate (Figure S5A; Table S1).

Concerning the nucleophile tyrosine, Int contains two tyrosine residues located near the active site: Y379 at the end of αL points toward the scissile phosphate, and Y380 in the subsequent loop that threads away from the active site sits in a snug pocket on the interface of the two protein subunits (Figure 4A). Hydrophobic interactions and hydrogen bonds keep Y380 in a buried position, where it is unable to act as a nucleophile in DNA cleavage. Notably, Y379 is also relatively distant from the scissile phosphate in both active sites (P – OH distance: 4.9–5.4 Å).

We analyzed the presence of the two consecutive tyrosines in CTn transposases (sequences as in Figure S2D) and canonical tyrosine recombinases. Transposases of Tn*916*-like CTns usually contain two tyrosines, whereas only one tyrosine is found in other members of the superfamily (Figure 4B). The residues forming Y380’s binding pocket are also conserved within the Tn*916* family. To explore the two tyrosines’ roles in DNA cleavage and recombination, we generated single and double point mutants of Int^82N^, substituting one or both with phenylalanine that cannot cleave DNA. Both single mutants cleaved DNA *in vitro*, whereas the double mutant was completely inactive (Figure S5B). However, in strand exchange Y380F was completely inactive, whereas Y379F was active as the unmutated protein. Thus, while both Y379 and Y380 can perform DNA cleavage, only cleavage via Y380 can proceed to strand exchange. In agreement, *in vivo* assays in *E.coli* showed that Y380 is critical for Tn*1549* excision, whereas Y379 is dismissible (Lambertsen et al., 2018).

### DNA Cleavage Occurs inside the Transposon IR

Sequencing of *in vivo* Tn*1549* intermediates and integration sites previously implied that CI cleavage occurs at the IR boundary (Lambertsen et al., 2018, Launay et al., 2006), ensuring that the entire transposon sequence is strictly preserved during transposition. In contrast, in the Int-CI complexes, the catalytic residues are packed around the phosphate groups 1 nt inside the transposon end (position T-1/T-1′ in IR_L_/IR_R_; Figures 4A and S5A).

To determine the exact cleavage position, we used phosphorothioate (PTO) mapping (Ennifar et al., 2003). Strand exchange assays with PTO 1nt inside the IR (T-1/T-1′) strongly inhibited Int activity, whereas PTO at the IR boundary (*a*0/*g*0′) had much less effect (Figure 4C). This allocated the cleavage position to T-1/T-1′ inside the transposon end as indicated by our structures. In IR_L_, we observed residual activity with PTO at T-1 and some inhibition with modified *a*0, indicating some cleavage ambiguity at this transposon end. Mapping at IR_R_ was unambiguous and results were consistent independent of the length of the crossover region (Figures 4C and S5C).

### Subterminal Cleavage Is Required for Efficient Strand Exchange

To explore the importance of the cleavage position for subsequent strand exchange, we next tested the efficiency of Int-mediated DNA ligation with “half-site” substrates. These contain only one IR site and allow to specifically monitor ligation with another pre-cleaved half-site substrate (see schematics in Figure 4D) (Nunes-Düby et al., 1989). We only observed ligation with partner substrates that contain the terminal T-1/T-1′ nucleotide in the exchanged strand at both IRs (Figure S5D). Thus, cleavage inside the transposon IR is essential for productive ligation during strand exchange.

During strand exchange, the liberated 5′OH groups need to be placed precisely in the active sites of their partner strands, so that ligation can occur (Figure 1B). Thus, our finding that CI cleavage leaves a conserved thymine base at the 5′OH on the cleaved strand and an unpaired adenine base on the uncleaved strand in the Int active site, together with the fact that the Tn*1549* target sites are AT-rich (Lambertsen et al., 2018, Launay et al., 2006) led us to ask if base pairing at the ligation site helps to promote strand exchange. Ligation assays with partner substrates containing different bases at the 5′OH revealed product only with thymine (Figure 4D). Ligation was abolished by any mutation of the terminal base in either IR_L_ or IR_R_, irrespective of the length of the crossover region (Figure S5E), showing that this base plays a critical role in strand exchange.

The structurally observed melting of the CI DNA suggested that short single-stranded DNA (ssDNA) oligonucleotides that can intercalate in the opened crossover region would block strand exchange by interfering with recruitment of the exchanged strand. To check this, we used 5- and 10-mer ssDNA molecules with a sequence representing the crossover region, ending with a 5′T. Addition of increasing amounts of ssDNA gradually inhibited strand exchange, with a complete loss of the expected product at ∼1.7 μM (Figure S5F).

### Int Resolves the HJ Intermediate

**Figure 5:**
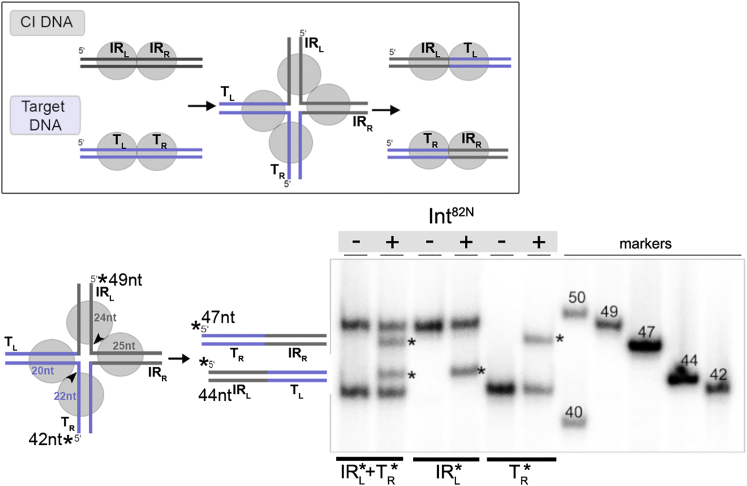
Int Resolves the HJ Intermediate Schematics of the HJ and its resolution. Results of HJ resolution assays with IR_L_ and/or T_R_ arms radiolabeled (^∗^) on denaturing PAGE gel. Int^82N^ -mediated cleavage and strand exchange (at the arrowheads) leads to the final integration products (IR_L_-T_L_, 44 nt and T_R_-IR_R_, 47 nt). See also Figure S6.

**Figure S6:**
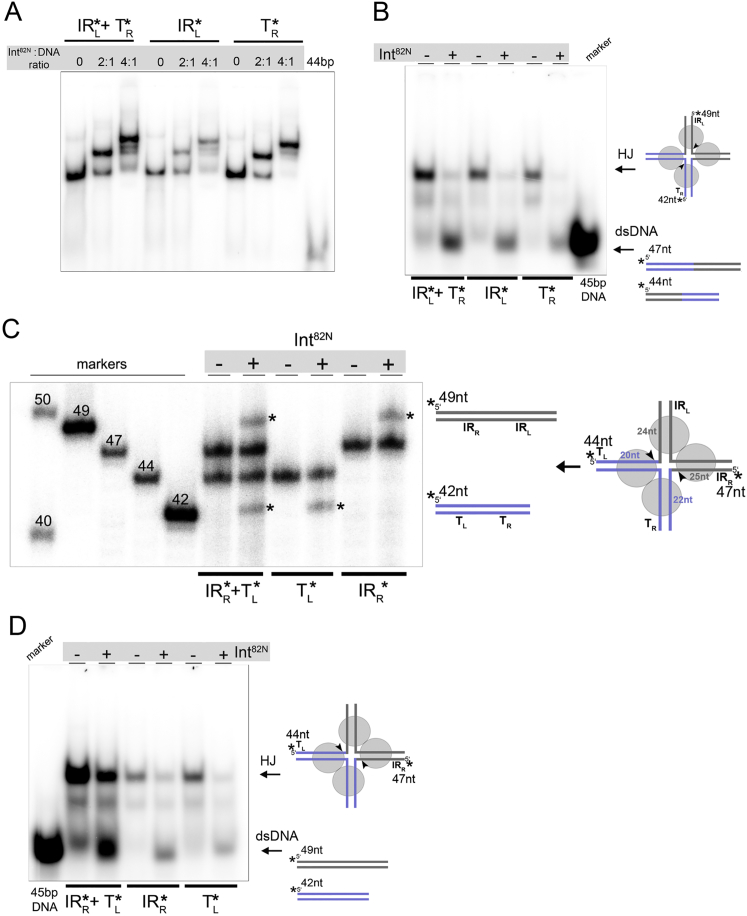
Characterization of Int’s HJ Resolution Activity, Related to Figure 5 (A) Int^82N^ binds to the synthetic HJ, forming stable complexes at 4:1 ratio. HJ substrates were radioactively labeled at the IR_L_ or T_R_ arm or both (see schematics in Figure 5). Complexes were prepared at a constant concentration of radiolabeled HJ (1 μM) and different protein concentrations (2 and 4 μM), and run on a native PAGE gel (6% TBE gel). 44bp dsDNA is shown as a control and migrates faster than the HJ. (B) Int^82N^ resolves HJ intermediates toward recombined products. Native gel with the purified radiolabeled DNA products of the HJ resolution assay shown in Figure 5. Reactions with (+) and without (-) Int^82N^ confirm that Int^82N^ has resolved the HJ to dsDNA. (C) Int^82N^ can resolve HJ intermediates back to substrates. HJ was radioactively labeled on the IR_R_ arm, the T_L_ arm or both as indicated (stars). Solid arrowheads mark the cleavage positions. The sizes of the individual arms, as well as the lengths of the substrates (44 nt and 47 nt) and recombinant products (42 nt and 49 nt) are indicated in the schematics. Reactions with (+) or without (-) Int^82N^ are visualized on a 12% TBE-Urea gel. Upon Int^82N^ activity, the sizes of the labeled strands are changed to the expected product sizes. Size markers (49, 47, 44 and 42 nt) are shown on the left. (D) Native gel with the purified DNA products from C confirms HJ resolution to dsDNA substrates.

Our structures showed that the Int-CI complex contains a transposase dimer bound to a straight DNA substrate in a pre-catalytic conformation. In contrast, active structures of four other tyrosine recombinases revealed tetrameric complexes with two largely bent DNA molecules (Figure S1F). Thus, we asked if the downstream steps of the Int-mediated reaction follow the canonical pathway of tyrosine recombination. We tested if Int could assemble on a four-way HJ DNA intermediate and resolve this correctly into recombination products. The HJ substrate mimicked the intermediate formed between the CI and a previously observed integration site (Lambertsen et al., 2018) (Figure 5). Int^82N^ formed stable complexes with the HJ at 4:1 ratios (Figure S6A). We monitored cleavage and rejoining by differential labeling of each distinctly sized DNA arm. Int^82N^ joined IR_L_ to the left side of the integration target site (T_L_) and IR_R_ to the right side of the target (T_R_), demonstrating that it can resolve the HJ intermediate to recombined dsDNA products (Figures 5 and S6B). Notably, resolution was also observed in the opposite direction, back to the original substrates (CI and target site; Figures S6C and S6D). These results indicate that Int can form active tetramers that are competent in HJ resolution.

### C-Terminal Truncations Enhance Cleavage and Strand Exchange

**Figure 6:**
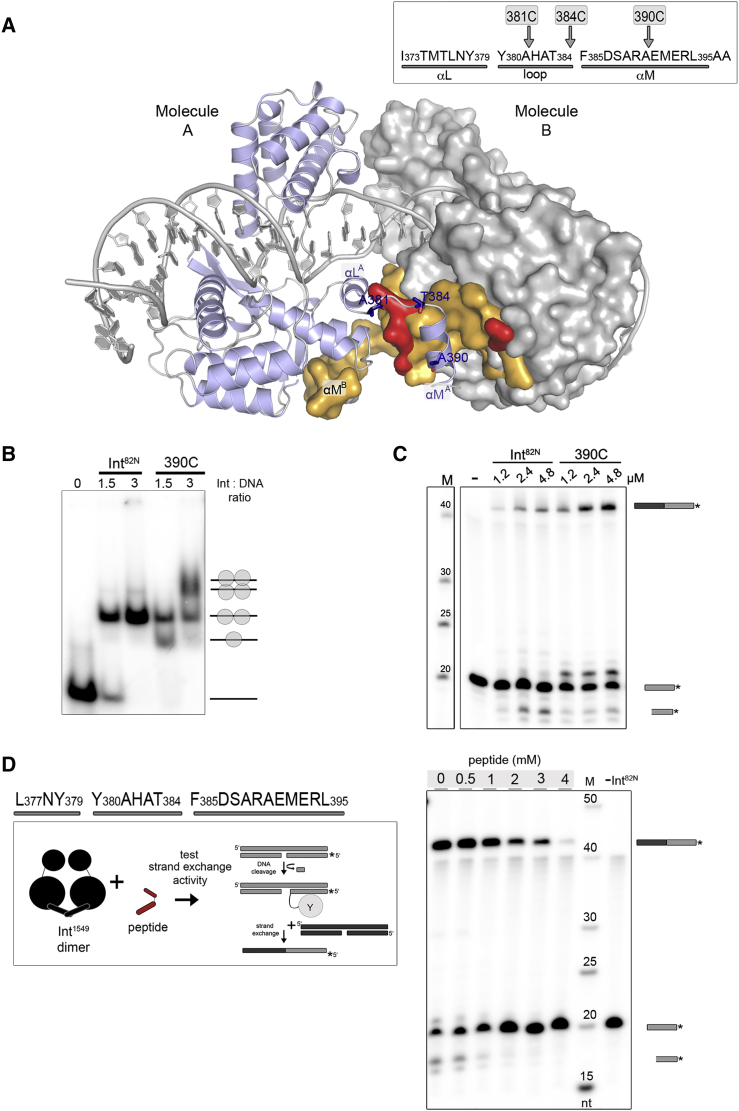
The Role of Int’s C-Terminal Helix (A) The Int^82N^ dimer interface. Molecule B is shown as surface (gray); residues interacting with the C-terminal helix of Molecule A (blue) are colored: orange, hydrophobic; red, hydrophilic interaction (see Table S5). C-terminal truncations are marked. (B) 390C forms higher oligomers with CI5 DNA on EMSA. Schemes indicate putative complex stoichiometries. (C) 390C is hyperactive in strand exchange. Superfluous lanes were removed. (D) Synthetic peptide antagonizes Int activity. Strand exchange reactions with different peptide amounts (at constant 33 μM Int^82N^ and 1 μM ^32^P-labeled CI5 DNA). Cleavage and recombination products decrease gradually. See also Figure S7.

**Figure S7:**
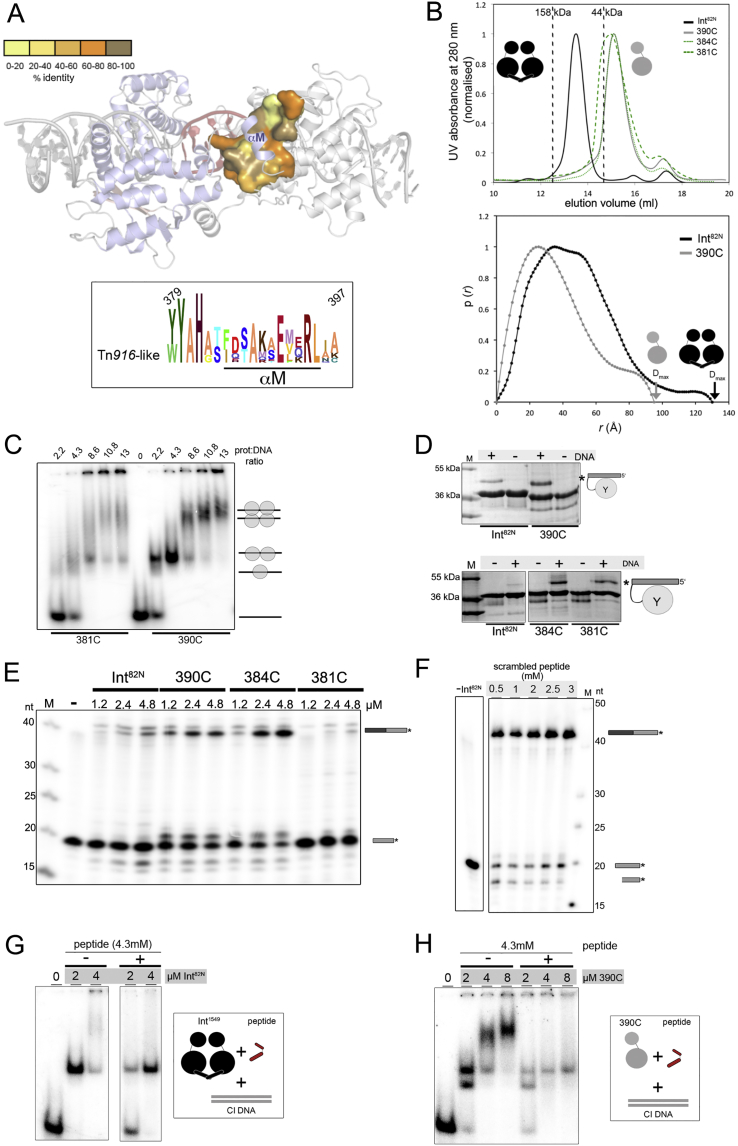
Int’s C-Terminal Helix Mediates Protein Oligomerization and Catalytic Activity, Providing a Target for Inhibitor Design, Related to Figure 6 (A) Conservation of the C-terminal helix αM and its binding site in Tn*916*-like CTn transposases. *Top*: Surface residues interacting with αM in the Int^82N^ dimer are colored by conservation (exact % identity values are listed in Table S5). *Bottom*: Sequence LOGO for αM. (B) Size exclusion chromatography (SEC, *top*) and small angle X-ray scattering (SAXS, *bottom*) reveal dimers for Int^82N^ and monomers for C-terminally truncated variants. In SEC, the elution profiles for Int^82N^ (black line), 390C (gray line), 384C (green dots) and 381C (green dashes) are compared on Superdex 200 10/300 column. C-terminally truncated variants show an elution volume corresponding to a molecular weight of ∼36 kDa, whereas Int^82N^ elutes as a dimer at ∼72 kDa. For SAXS, pairwise distance distribution function p(*r*) is plotted. Maximal particle dimensions (D_max_) are 95Å for 390C and 130Å for Int^82N^. The sharp drop in D_max_ determination indicates conformational flexibility (especially for the monomeric 390C construct; see STAR Methods for details), likely reflecting free movement of the two Int domains in absence of DNA. (C) C-terminally truncated Int variants form higher oligomers upon DNA binding. Complexes were prepared at a constant concentration of radiolabeled CI5 DNA with different concentrations of 381C and 390C and run on a native gel (TBE 4%–20% PAGE gel). The presumed protein-DNA composition is shown schematically for each band. (D) Truncated variants are hyperactive in DNA cleavage. Reactions with suicide CI5 DNA are analyzed on a 12% SDS-PAGE gel. Asterisk marks the covalent cleavage product. (E) 390C and 384C variants are hyperactive in strand exchange under low protein concentration. Strand exchange reactions with Int^82N^, 390C, 384C and 381C constructs are run on denaturing 12% TBE-Urea PAGE gel. The recombined product is indicated with a black/gray bar. The 381C construct shows a great reduction in strand exchange activity. (F) Strand exchange assays in the presence of a scrambled peptide do not show a dose dependent inhibition as seen for the native peptide (Figure 6D). Reactions with different peptide concentrations (as marked above the gel) at a constant concentration of protein (33 μM), radiolabeled suicide CI5 DNA (1 μM) and unlabeled DNA (75 μM) are visualized on denaturing 12% TBE-Urea gel. Products are marked with schematics. ‘-Int^82N^’, reaction without protein. (G) The C-terminal tail mimicking peptide affects Int^82N^-CI5 complex formation. Complexes were prepared with constant peptide and DNA concentration with different protein amounts (as marked). Complexes are run on a native gel (TBE 4%–12% PAGE). Addition of the peptide reduces protein-DNA complex formation. (H) The peptide compromises assembly of higher order Int-CI complexes. Complexes were prepared at constant concentration of the peptide and CI5 DNA with increasing concentrations of the C-terminally truncated 390C Int variant (as marked), and run on a native gel (TBE 4%–12% PAGE).

Next, we investigated the role of the structurally observed dimeric assembly in transposition, and the impact of the C-terminal helix in stabilizing the dimer. Int’s αM sits in a groove on the surface of its partner subunit, creating a large part of the dimer interface via mostly hydrophobic contacts (82%; Figure 6A; Table S5). The sequence of αM and its docking site are well conserved among Tn*916*-like transposases (Figure S7A). We made serial truncations of the C terminus in Int^82N^ (390C, 384C, 381C) and tested their effect on dimerization and activity. All constructs were monomeric in analytical size exclusion chromatography, showing that removing just 7 amino acids (as in 390C) is enough to disrupt the dimer (Figure S7B). Small angle X-ray scattering data confirmed these results, revealing monomers for 390C (estimated Mw 30.6 ± 1.9 kDa), whereas the intact Int^82N^ showed a dimeric state (Mw 66.5 ± 8.6 kDa; Figure S7B).

All constructs bound DNA, but the cooperativity of DNA binding and the oligomeric state of the complexes were different (Figures 6B and S7C). While the dimeric Int^82N^ formed 2:1 protein-DNA complexes, the C-terminally shortened variants also formed monomeric and higher order oligomeric complexes that migrated consistent with a 4:2 tetrameric assembly. The lack of these assemblies with Int^82N^ is consistent with a role for the C-terminal helix in stabilizing a pre-synaptic dimer. 390C and 384C showed higher DNA cleavage and strand exchange activity than Int^82N^, whereas the 381C variant was reduced in strand exchange (Figures 6C, S7D, and S7E). Thus, deletion of the entire αM helix compromises Int function, but small C-terminal truncations can promote synapsis and activity, probably by destabilizing the pre-catalytic dimer conformation.

### A Peptide Antagonist Blocks Int Activity

Because inhibiting conjugative transposition may provide a strategy to limit antibiotic resistance spreading, the observed critical role of Int’s C-terminal helix led us to assess whether allosterically blocking Int oligomerization affects transposition activity. For a proof of principle, we designed a peptide covering 19 residues of Int’s C terminus and tested its ability to alter Int activity *in vitro*. Peptide addition resulted in a prominent dose-dependent decrease of cleavage and recombination, with ∼4 mM of the peptide completely blocking Int^82N^ activity (Figures 6D and S7F). This inhibitory effect was concomitant with a reduction in DNA binding and complex oligomerization (with Int^82N^ and 390C, respectively; Figures S7G and S7H). These results strongly suggest that the peptide blocks Int function by mitigating essential protein-protein interactions during functional protein-DNA complex assembly.

## Discussion

### Model for Tn*1549* Integration

**Figure 7:**
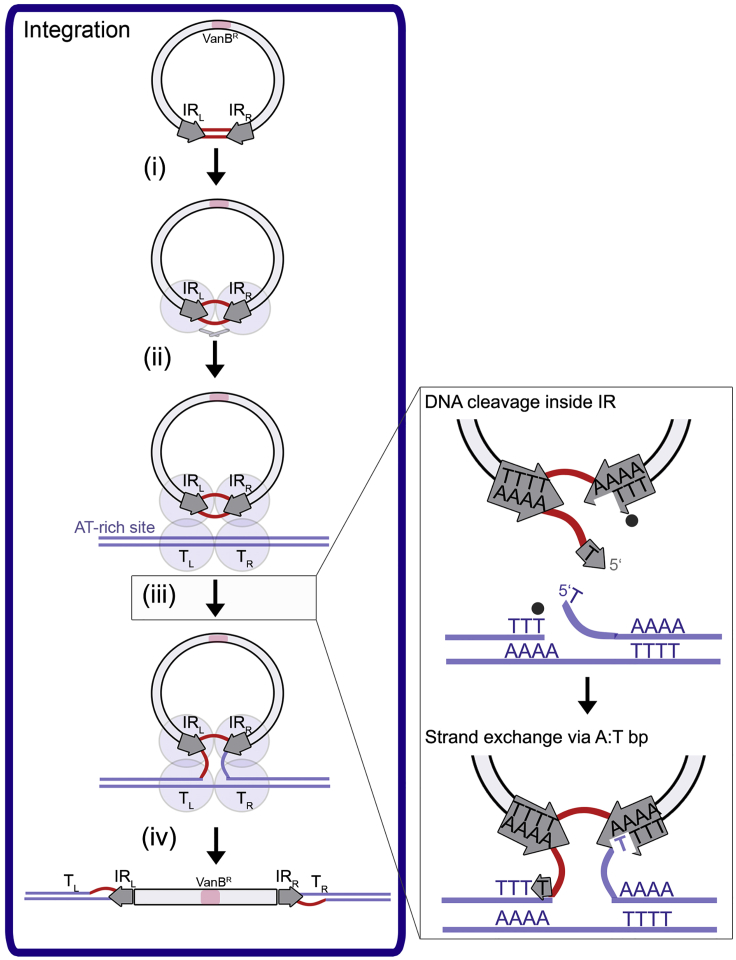
Model for Tn*1549* Integration Int (gray ovals) binds to CI DNA (black circle) as a dimer and actively opens the crossover region (red) (i). Int is in an inactive conformation. Synapsis between CI and target DNA (blue) captures an AT-rich chromosomal site (ii). Following structural rearrangement and activation, Int-mediated cleavage and ligation of one strand pair generates an HJ intermediate (iii). Int cleaves 1nt inside the transposon end (top inset) and gets covalently attached to the IR DNA (black dot). The target site is cleaved at a conserved T. The strands are exchanged; base pairing at the 5′Ts helps join the partner strands (bottom inset). HJ resolution integrates Tn*1549* in the recipient genome (iv). Again, Int cleaves upstream of a conserved T in the CI and the target and base pairing promotes strand ligation. See also Movie S1.

From our structural results, together with biochemical and microbiological data, we propose the following model for Tn*1549* integration (Figure 7).

First, Int binds as a dimer to the CI (Figure 7, step i), recognizing the transposon ends mainly via indirect sequence readout. Int’s essential β-hairpin senses the IR DNA shape and N150 specifically locates the terminal bases. Upon binding, Int actively distorts the DNA duplex at the crossover region by base flipping (via R153) and unwinding. This introduces flexibility in the DNA, allowing Int to accommodate diverse transposon CIs. In the Int-CI complex, the active sites are arranged at the scissile phosphates near the IR boundaries, but they are incompletely assembled. A major conformational change probably occurs upon synapsis with target DNA, which brings the nucleophile tyrosine into an activated position where it can attack the DNA (Figure 7, step ii).

Following activation, Int cleaves one DNA strand, resulting in a 3′-phosphotyrosine bond with the IR DNA. The terminal IR base (T-1) remains attached to the crossover DNA carrying the liberated 5′OH group (Figure 7, step iii, top insert).

Next, the 5′OH of the CI is exchanged with target DNA and attack of the target 5′OH resolves the Int-CI linkage and ligates the DNA. Simultaneously, the CI 5′OH is joined to its partner strand in the target, generating a HJ with one transposon strand fully fused to the target DNA (Figure 7, step iii, bottom insert). Protein-induced opening of the DNA duplex at the crossover region promotes strand exchange, as it allows to *a priori* melt the complementary DNA strands at the crossover region, helping to overcome the energy barrier for strand exchange. Base pairing of the exchanged T-1 with an unpaired adenine base in the partner substrate aids recruitment and ligation of the 5′OH. In agreement, we have recently shown that Tn*1549* integration sites have a consensus sequence of TTTT-n6-AAAA *in vivo* (Lambertsen et al., 2018), so that target DNA generally contain adenines that can pair with T-1 from the transposon CI. This proposed reaction scheme is supported by three previously unexplainable aspects of conjugative transposition: (1) the tolerance for low sequence homology between the recombination substrates (i.e., the transposon flanks or the CI and the target), (2) the variety of naturally occurring active CIs, and (3) the requirement for AT-rich integration sites.

Last, the four-way CI-target HJ is resolved through cleavage and strand exchange of the second strand pair (Figure 7, step iv). Cleavage again occurs 1 nt inside the respective IR and base pairing at the ligation site guides strand exchange, joining the second CI strand to the target DNA and creating the final integrated product.

We propose that transposon excision follows a similar pathway, involving recombination of the two transposon ends by cleavage 1 nt inside the IR and 7 nt into the flanking DNA. This results in 8 nt stagger on both transposon ends that—due to strict conservation of the terminal bases—generate a CI with 6 bp heteroduplex. In agreement, we have recently shown that T:A at position 7 in the left flank is critically important for excision *in vivo* (Lambertsen et al., 2018).

### The Int^82N^-CI Complex Is an Auto-Inhibited Preparatory Step for Integration

Unlike other tyrosine recombinase structures that contain four protein and two DNA molecules in a synaptic complex, the Int^82N^-CI complex is dimeric with two proteins bound to one DNA. The interface between the Int subunits is more hydrophobic than the ones observed in previous tyrosine recombinase structures (82% compared to e.g., 44% for the λ phage integrase), allowing Int to form stable dimers by itself while others are monomeric without DNA (Lee et al., 2004). Instead, the Int dimer conformation is reminiscent of the DNA-free structure of the HP1 integrase catalytic domain (Hickman et al., 1997) that was proposed to rearrange upon DNA binding. The dimer brings a striking parallel with DNA complex structures of distantly related protelomerases (Aihara et al., 2007, Shi et al., 2013) that maintain the telomeres of bacteria with linear chromosomes. They cleave DNA as tyrosine recombinases, but cut both strands of a single DNA duplex at once in an interlocked dimer, distorting the DNA to promote formation of hairpin telomeres after replication (Kobryn and Chaconas, 2014). However, Int dimers are locked in an inactive conformation, implying that they represent a regulatory intermediate that is not yet licensed for recombination. Consistent with this idea, destabilization of the dimer by shortening the C terminus facilitated oligomerization (synapsis) of Int-DNA complexes and led to hyperactivity *in vitro*. Thus, we conclude that the Int^82N^-CI structure represents an auto-inhibited pre-synaptic intermediate, with the Int C-terminal tail holding the enzyme in an inactive conformation. While the C-terminal segments regulate activity of various tyrosine recombinases by positioning the catalytic tyrosine (Grindley et al., 2006), a role in locking inactive protein-DNA complexes was not seen before. The auto-inhibited state may help to prohibit futile cleavage of the transposon CI until a suitable target DNA is found and successful integration can occur.

### Conformational Rearrangements upon Target Binding

Target DNA capture by the Int-CI complex probably occurs in a tetrameric synaptic complex, where a second pair of Int subunits holds the target. We envision that two DNA-bound dimers first come together to assemble a tetramer with straight DNA, which then undergoes activation by DNA bending to create an arrangement similar to active structures of other tyrosine recombinases (Biswas et al., 2005, Guo et al., 1997). This mechanism is supported by the fact that our oligomerization antagonist peptide blocks DNA cleavage and by studies of the XerH-*dif*_*H*_ complex, which showed that synaptic tetramers first assemble in a pre-catalytic state with straight DNA and are then activated via structural rearrangement and DNA bending (Bebel et al., 2016). As shown on Movie S1, the conformational changes required for tetramerization are straightforward to visualize based on the λInt-COC′ synaptic complex structure by modeling. The modeled tetramer is held together by a large protein interface (1,393 Å^2^; PISA) involving interactions of CB, CAT, and the circularly swapped C-terminal helices. In the natural context, additional players (such as the AB domain or accessory protein factors) may help promote tetramerization and activation as proposed for λ and Tn*916* (Rudy et al., 1997, Warren et al., 2005). The notion of a synaptic tetramer is also supported by the fact that Int resolves preformed HJ substrates. Here, external components may also help to shape the synaptic complexes, so as to drive the reaction efficiently toward products.

### Tyrosine Recombination without Homology in Tn*916*-like Transposons

Whereas most transposon families move via linear DNA intermediates that are cut out and integrated by RNaseH-type nucleases (i.e., DDE-transposases), CTn transposases recombine transposon ends without double strand breaks generating a sealed CI that is fairly stable and can cross cellular boundaries. Perhaps the most puzzling question about transposition of Tn*916*-like CTns was how their transposases could excise and integrate the transposon without clear sequence homology between the DNA substrates. Our biochemical and structural data on Tn*1549*, a prominent member of the family, now reveals surprising answers.

First, we have discovered that the Tn*1549* Int actively melts the DNA at the non-homologous crossover region, thereby obviating the need for extended sequence homology for strand exchange. As diverse CIs and integration sites are common for all studied Tn*916*-like transposons (Caparon and Scott, 1989, Launay et al., 2006) and the protein residues required for DNA melting are conserved, this mechanism is likely shared across the Tn*916*-like family. The use of DNA distortions to promote DNA rearrangements also appears to be a reoccurring theme in transposition. Various DDE transposases use base flipping to promote e.g., second strand cleavage by hairpin formation in Tn*5*/*10* (Bischerour and Chalmers, 2007) or target DNA recognition and integration in Mos1 (Morris et al., 2016). Our results demonstrate that tampering with the DNA duplex structure also plays a critical role in the function of the structurally and mechanistically unrelated Tn*916*-like Y-transposases.

Second, we show that cleavage in the transposon ends creates a single base pair homology allowing the exchanged DNA to find their partner strands. Consistently, work on Tn*916* indicated cleavage inside the transposon ends (Taylor and Churchward, 1997) and conservation of TA pairs flanking a 6 bp variable region in the target sites (Lu and Churchward, 1995), supporting the idea that a single base pair is sufficient to promote transposition of Tn*916*-like elements in general. This absolute minimum homology requirement is even less than the 2 nt conservation observed for the *Bacteroides* CTnDOT (Laprise et al., 2010) and appears to uniquely allow Tn*916*-like Y-transposases to overcome the gross lack of DNA homology between their substrates.

The structural plasticity of the DNA can also explain how CIs with different lengths arise during transposon excision, as flexibility may allow the backbone to slide within the Int active site, resulting in cleavage at diverse positions. This is consistent with previous work on Tn*916* showing low levels of alternative cleavage leading to a variety of CIs (Taylor and Churchward, 1997).

Another feature of Tn*916*-like Y-transposases is the presence of two consecutive tyrosines near the active site. Their intriguing biochemical duality and high conservation point toward distinct biological functions. It seems possible that Y379 allosterically blocks Y380 from entering the active site helping to stabilize the auto-inhibited conformation prior to target capture, or the two tyrosines may both act as nucleophiles in different stages of transposition as seen for conjugative relaxases (Dostál et al., 2011).

Finally, our discovery of the C terminus-mediated auto-inhibitory mechanism proposes a strategy to allosterically inhibit Tn*1549* Int activity. As the protein segments involved are conserved within the Tn*916*-like CTn family (Figure S7A), but absent in other tyrosine recombinases (Figure 4B), our peptide antagonist may provide a broadly applicable approach against Tn*916*-like elements, while not affecting cellular recombinases or other integrases.

As Tn*916*-like conjugative transposons provide a major driver for horizontal transfer of antibiotic resistance genes across diverse bacteria, their targeting could open new possibilities for limiting resistance dissemination and multidrug-resistance. By presenting the first high resolution structure of a CTn system and demonstrating its value for designing strategies to block transposition, our study takes the first step in this direction. Using two alternative approaches, (1) with a peptide that allosterically blocks the protein interface seen in our structures, and (2) with DNA oligonucleotides that anneal to the melted DNA blocking strand exchange, we show that interfering with the transposase-DNA complex architecture leads to transposition inhibition. As our structural findings appear to be of general relevance to the Tn*916*-like CTn family, it will be interesting to determine if similar approaches can be used to limit transposition for other family members. If so, our results could provide more widely applicable possibilities for controlling antibiotic resistance spreading.

## STAR★Methods

### Key Resources Table

**Table:** 

REAGENT or RESOURCE	SOURCE	IDENTIFIER
**Bacterial and Virus Strains**		

*E. coli* BL21(DE3) pLysS	Novagen	Cat#69451
*E. coli* Top10 (recA-)	Thermo Fisher	Cat#C404003

**Chemicals, Peptides, and Recombinant Proteins**		

Phusion Flash High-Fidelity Master Mix	Thermo Fisher	Cat#F548
DNase	Roche	Cat#10104159001
cOmplete EDTA-free	Roche	Cat#11873580001
HisTrap HP	GE Healthcare	Code#17-5248-02
SenP2 protease	PepCore, EMBL	N/A
Superdex 200 10/300	GE Healthcare	Code#17517501
24 well sitting drop plate (Cryschem Plate)	Hampton Research	Cat#HR3-158
[γ-^32^P]-ATP	Hartmann	Cat#SRP-301
T4 polynucleotide kinase	NEB	Cat#M0236L
Micro Bio-Spin 6 Columns	Bio-Rad	Cat#732-6221
4-12%TBE Gels, 12 well	Thermo Fisher	Cat#EC62352BOX
4-20% TBE Gels 1.0 mm, 12 well	Thermo Fisher	Cat#EC62252BOX
SYBR Gold	Thermo Fisher	Cat#S-11494
Coomassie G250 – Brilliant Blue G250	Sigma Aldrich	Product#27815-25G-F
Mark12 Protein Standard	Thermo Fisher	Cat#LC5677
Proteinase K	Carl Roth	Cat#7528.1
10/60 Ladder	IDT	Cat#51-05-15-01
HyperLadder 1kb	Bioline	Cat#BIO-33053
Peptides (specific)	ProteoGenix SAS	N/A
LNYYAHATFDSARAEMERL		
Peptides (scrambled)	ProteoGenix SAS	N/A
LYDLFAAHAEMARYNSTRE		

**Deposited Data**		

Atomic model of Int^82N^(R225K)-CI5 complex	This study	PDB: 6EMZ
Atomic model of Int^82N^(R225K)-CI6a complex	This study	PDB: 6EN1
Atomic model of Int^82N^(R225K)-CI6b complex	This study	PDB: 6EN2
Atomic model of Int^82N^(wt)-CI5 complex	This study	PDB: 6EN0
Atomic model of Int^82N^(Y379F)-IR_R_ complex	This study	PDB: 6EMY

**Oligonucleotides**		

PCR primers (Table S6)	IDT	N/A
Crystallization oligonucleotides (Table S6)	IDT	N/A
DNA oligonucleotides for cleavage and strand exchange assays (Table S6)	IDT	N/A
DNA oligonucleotides with phosphorothioate (PTO) modification (Table S6)	IDT	N/A
Half-site oligonucleotides with truncated variants (Table S6)	IDT	N/A
Half-site oligonucleotides (Table S6)	IDT	N/A
DNA oligonucleotides for crossover competition assays (Table S6)	IDT	N/A
DNA oligonucleotides for Holliday Junction resolution assays (Table S6)	IDT	N/A
DNA oligonucleotides with 2-aminopurine (2AP) modification (Table S6)	IDT	N/A
DNA oligonucleotides for *in vivo* assays (Table S6)	IDT	N/A

**Recombinant DNA**		

Plasmid: pETM28	PepCore, EMBL	N/A
Plasmid: pETM28-Int^FL^	This study	N/A
Plasmid: pETM28-Int^FL^ (R225K)	This study	N/A
Plasmid: pETM28-Int^82N^	This study	N/A
Plasmid: pETM28-Int^82N^ (R225K)	This study	N/A
Plasmid: pETM28-Int^82N^ (R153A)	This study	N/A
Plasmid: pETM28-Int^82N^ (Y160A)	This study	N/A
Plasmid: pETM28-Int^82N^ (R153-Y160/A)	This study	N/A
Plasmid: pETM28-Int^82N^ (Y160A)	This study	N/A
Plasmid: pETM28-Int^82N^ (390C)	This study	N/A
Plasmid: pETM28-Int^82N^ (384C)	This study	N/A
Plasmid: pETM28-Int^82N^ (381C)	This study	N/A
Plasmid: pKK223-3 containing the mini-Tn*1549* transposon [Cm^R^]	Lambertsen et al., 2018	N/A
Plasmid: pKK223-3 containing the mini-Tn*1549* transposon and Gm resistance on the backbone [Cm^R^/Gm^R^]	Lambertsen et al., 2018	N/A
Plasmid: pBAD-Xis/Int^FL^ [Ap^R^]	Lambertsen et al., 2018	N/A
Plasmid: pBAD-Xis/Int^FL^(R225K) [Ap^R^]	This study	N/A
Plasmid: pBAD-Xis/Int^FL^(R153A) [Ap^R^]	This study	N/A
Plasmid: pBAD-Xis/Int^FL^(R153A-Y160A) [Ap^R^]	This study	N/A

**Software and Algorithms**		

XDS	Kabsch, 2010	http://xds.mpimf-heidelberg.mpg.de/
Phaser	Adams et al., 2010	https://www.phenix-online.org/
PHENIX	Adams et al., 2010	https://www.phenix-online.org/
COOT	Emsley et al., 2010	http://www2.mrc-lmb.cam.ac.uk/personal/pemsley/coot/
Mafft	Katoh and Standley, 2013	https://www.ebi.ac.uk/Tools/msa/mafft/
PhyML	Guindon et al., 2010	http://www.atgc-montpellier.fr/phyml/
ProtTest	Abascal et al., 2005	http://darwin.uvigo.es/software/prottest_server.html
aBayes	Anisimova et al., 2011	http://www.atgc-montpellier.fr/phyml/versions.php
PRIMUS	Konarev et al., 2003	https://www.embl-hamburg.de/biosaxs/primus.html
FATCAT	Li et al., 2006	http://fatcat.sanfordburnham.org
Phyre2	Kelley et al., 2015	http://www.sbg.bio.ic.ac.uk/∼phyre2/
HADDOCK	van Zundert et al., 2016	http://milou.science.uu.nl/services/HADDOCK2.2/
3DNA	Lu and Olson, 2003	http://x3dna.org/
PyMOL	Schrödinger	https://pymol.org/2/
DALI	Holm and Rosenström, 2010	http://ekhidna.biocenter.helsinki.fi/dali_server/start
ESpript	Robert and Gouet, 2014	http://espript.ibcp.fr/ESPript/ESPript/
PISA	Krissinel and Henrick, 2007	http://www.ebi.ac.uk/pdbe/pisa/
NUCPLOT	Luscombe et al., 1997	https://www.ebi.ac.uk/thornton-srv/software/NUCPLOT/
ImageQuantTL v8.1.0.0	GE Healthcare	Code#29000605

**Other**		

WEBlogo server	University of California, Berkeley	http://weblogo.berkeley.edu/
Phosphoimager Typhoon FLA 7000	GE Healthcare	Code#28955809
10 × 2 mm light path cuvettes	Hellma Analytics QS	Article ID: 101-015-40
PTI QuantaMaster 8000 Fluorometer	Photon Technology International	N/A
GenScript server	N/A	https://www.genscript.com/peptide_screening_tools.html

### Contact for Reagent and Resource Sharing

Further information and requests for resources and reagents should be directed to and will be fulfilled by the Lead Contact, Orsolya Barabas (barabas@embl.de).

### Experimental Model and Subject Details

For *in vitro* studies and crystallography, Int variants were produced in *Escherichia coli* BL21(DE3)pLysS cells (Novagen) grown in Luria-Bertani (LB) medium. Expression was induced with 1 mM isopropyl β-D-1-thiogalactopyranoside (IPTG, final concentration) when the bacteria density reached an OD600 of 0.6-0.8, and the cells were grown for 24h at 180 rpm at 18°C.

For *in vivo* transposon excision assays, *E. coli* Top10 cells (recA-, Thermo Fisher) were grown in Luria–Bertani (LB) medium with shaking at 150-170 rpm at 37°C. When the strains contained a protein expression plasmid (pBAD, see Method Details) a final concentration of 0.2% glucose (D-glucose, Sigma-Aldrich) was also added to the media to repress leaky protein expression. Protein expression and transposition was then induced with 0.2% arabinose (L-arabinose, Sigma-Aldrich, final concentration).

### Method Details

#### DNA constructs and oligonucleotides

The DNA encoding for Int^FL^ from *Enterococcus faecalis* (GENEBANK: AAF72368.1) was synthesized with codon-optimization for expression in *E. coli* (GeneArt, Thermo Fisher). The Int^FL^ and Int^82N^ constructs were cloned into the expression vector pETM28 (PepCore, EMBL) by restriction cloning using the BamHI/XhoI restriction sites. The mini-Tn*1549* donor plasmid (DP, pKK223-3 derivative) and the wild-type pBAD-Xis/Int^FL^ protein expression plasmid (pBAD derivative encodig Tn*1549* Xis and Int^FL^) were obtained from (Lambertsen et al., 2018). All other plasmid constructs were prepared by site-directed mutagenesis using PCR primers listed in Table S6 with Phusion Flash High-Fidelity PCR Master Mix following the manufacturers’ instructions (Thermo Fisher).

DNA oligonucleotides were synthetized by Integrated DNA Technologies (IDT), purified via standard desalting for *in vitro* assays or polyacrylamide gel electrophoresis (PAGE) for crystallization, and their sequences are listed in Table S6.

#### Protein expression and purification

For recombinant protein production, *E. coli* BL21(DE3)pLysS cells were transformed with the respective pETM28-Int plasmid by electroporation. All Int variants were expressed as N-terminal fusions with hexa-histidine and SUMO tags in *E. coli* BL21(DE3)pLysS in LB medium at 18°C for 24h, after induction with 1 mM IPTG. Cells were lysed by sonication in Buffer A (50 mM HEPES pH 7.5, 750 mM NaCl, 50 mM imidazole, 1 mM DTT, with 0.05 mg/mL DNase, cOmplete EDTA-free protease inhibitor cocktail, Roche). The lysate was cleared by centrifugation at 20,000 g at 4°C for 30 min. The protein was purified via Ni-affinity chromatography by applying the soluble fraction to a Ni-Sepharose column (HisTrap HP; GE Healthcare). The protein was eluted with an imidazole gradient (50-500 mM in Buffer A) and the fractions containing the protein (elution range 162.5-275 mM imidazole) were pooled. The protein was then incubated with SenP2 protease (1:500) for 24h at 4°C in a Buffer C (50 mM HEPES pH 7.5, 250 mM NaCl, 5 mM DTT and 10% glycerol) to remove the 6xHis-SUMO tag, and further purified by size exclusion chromatography on a Superdex 200 10/300 column (GE Healthcare). The seleno-methionine derivative of Int^82N^ was expressed in M9 growth medium supplemented with the essential amino acids, with seleno-methionine replacing methionine, and was purified as above.

#### Crystallization and data collection

Whereas attempts to crystallize full-length Int^FL^ have thus far been unsuccessful probably due to the flexibility of the AB domain, we obtained high-quality crystals of Int^82N^ bound to CI DNA. Int^82N^-CI complexes were crystallized using 44bp and 45bp dsDNA oligonucleotides for CI5 and CI6, respectively. To avoid that DNA cleavage generates heterogeneity during crystallization, we used the catalytic mutant R225K. The analogous mutation was previously shown to block recombination without affecting the proper geometry of the active site in the Cre recombinase (Guo et al., 1997). DNA oligonucleotides were annealed in TE buffer (Tris-EDTA) at 500 μM concentration by heating to 98°C and slow cooling to room temperature. Complexes were formed by incubating Int^82N^ with DNA at a 2:1 molar ratio in Buffer C at 4°C overnight. All crystals were grown by vapor diffusion at 20°C in 24 well sitting drop plates (Hampton Research). Equal volumes (1 μL) of 10 mg/ml protein-DNA complex were mixed with well solutions (1 μL) and incubated against 500 μL of well solution as follows: for Int^82N^(R225K)-CI5 with 0.2M NH_4_-fluoride, 14% PEG3350, and for Int^82N^(R225K)-CI6a, Int^82N^(R225K)-CI6b and Int^82N^(wt)-CI5 with 0.1M Na-acetate pH 4.6, 30% PEG300. An Int^82N^(Y379F)-IR_R_ complex, containing half-site DNA was also crystallized using a well solution with 13% (v/v) PEG 3350, 0.25 M NaCl. Crystals were cryo-protected by transferring to well solution containing additional 10% glycerol or 12% (v/v) 2,3-butanediol (for the Int^82N^(Y379F)-IR_R_ complex) and flash frozen in liquid nitrogen. Diffraction data were collected on beamlines ID29, ID30A-1 and ID23-1 at the European Synchrotron Radiation Facility (ESRF) and on beamline P13 of PETRAIII/Deutsches Elektronen-Synchrotron (DESY, Hamburg). All datasets were processed with XDS (Kabsch, 2010).

#### Structure solution and refinement

All structures presented in this manuscript were solved by molecular replacement using Phaser in PHENIX (Adams et al., 2010). For Int^82N^(R225K)-CI5 and Int^82N^(R225K)-CI6a, the previously determined structure of an Int^82N^(Y379F)-IR_R_ complex (PDB: 6emy) was used as a search model. This model structure contained the Int^82N^ Y379F catalytic mutant in complex with a DNA substrate that represents only half of the Int^82N^ binding site (IR_R_) with a 6nt 5′ overhang, and was solved by single anomalous dispersion (SAD) method using anomalous data from a seleno-methionine derivative crystal in PHENIX Autosol (Adams et al., 2010) (see Table S1 for data collection and refinement statistics). For the Int^82N^(wt)-CI5 complex we used the Int^82N^(R225K)-CI5 structure, and for Int^82N^(R225K)-CI6b the Int^82N^(R225K)-CI6a structure as search model in the molecular replacement. After initial rigid body refinement of the solution with PHENIX, the full-length DNA could be easily seen in the electron density maps in all cases. The final models were obtained by alternating model building in COOT (Emsley et al., 2010) and simulated annealing (at the initial steps), restrained positional, TLS and ADP refinement in PHENIX (Adams et al., 2010). The data collection and refinement statistics are presented in Table S1. To reduce the effects of model bias and cross-validate DNA assignment, simulated annealed composite omit maps were calculated in PHENIX. The final structures include residues M82–A396 for CI5 and CI6a, and M82–L395 for CI6b. An additional residue, S81 is present due to SenP2 cleavage of the purification tags. Although a DNA length of 44-45bp was required to obtain good crystals, the electron density was weak at the ends and the terminal 2-4 bases could not be built. In the CI6a and CI6b structures the electron density was also unclear in the crossover region and some of the central nucleotides could not be built (see Figures 3 and S4).

#### Comparative analysis of the Tn*1549*-related integrases

First, we performed a blastp search against the UniprotKB database (http://www.uniprot.org/) using Tn*1549* Int as a query. The non-identical protein sequences exceeding soft criteria of 40% protein sequence identity to Tn*1549* Int were used in further analysis to ensure the inclusion of even distantly related sequences. This resulted in 359 distinct sequences present in 5379 genomic instances (as mapped by Identical Proteins Groups in NCBI, https://www.ncbi.nlm.nih.gov/ipg). Integrases of the SXT CTn, the Lambda and BPP-1 phages, as well as *E. coli* XerC and XerD were added to the dataset and used as an out group in the subsequent phylogenetic analysis. The sequences were then aligned using Mafft (Katoh and Standley, 2013) and the columns corresponding to the arm-binding domain or containing more than 90% gaps were removed. The phylogenetic tree was constructed by the PhyML package (Guindon et al., 2010) with LG+I+G model of protein evolution, evaluated by ProtTest (Abascal et al., 2005) and the statistical test of branch support was performed with aBayes (Anisimova et al., 2011). In the course of the reconstruction, we built 100 trees using both NNI and SPR moves and the tree with maximum likelihood value was used as the reconstruction of the phylogeny. The logos were produced using the web logo server (http://weblogo.berkeley.edu).

#### Preparation of radioactively labeled DNA substrates

DNA oligonucleotides were 5′ end labeled using [γ-^32^P]-ATP (Hartmann) and T4 polynucleotide kinase (NEB) and purified using Micro Bio-Spin 6 columns (Bio-Rad). The labeled DNA was mixed with the unlabeled complementary strand at a 1:5 molar ratio and annealed in annealing buffer (10 mM TrisHCl pH 8, 50 mM NaCl, 5 mM EDTA) by heating to 98°C for 2 min followed by slow cooling to 25°C. Suicide substrates, containing a nick in either DNA strand, were prepared by mixing an intact DNA strand with two short complementary strands and annealed as described above. Unlabeled DNA oligonucleotides used for strand exchange assays were phosphorylated at the 5′ end to avoid the production of aberrant products.

#### DNA binding assays

DNA binding was assessed using Electrophoretic Mobility Shift Assays (EMSA) with a constant 1 μM DNA concentration and increasing concentrations of protein as indicated in Figures 6, S3 and S7. All Int variants were incubated with DNA on ice for 20 min in Buffer C to allow complex formation. Following incubation, complexes were separated on non-denaturing polyacrylamide gels (4%–12% or 4%–20% TBE-PAGE gel, Thermo Fisher) using an electric field of 100 V/cm for 1h at room temperature. Bound and unbound DNA bands were visualized either using radioactively labeled (5′-^32^P) DNA in a Typhoon FLA 7000 phosphoimager (GE Healthcare) or by SYBR Gold (Thermo Fisher) staining of unlabeled DNA.

#### *In vitro* cleavage assays

Int cleavage activity was analyzed *in vitro* using ‘suicide’ CI DNA substrates (see schematics in Figure S1B for the assay design). These substrates contain a nick in the DNA backbone downstream of the expected cleavage position. Upon cleavage, 2 nucleotides diffuse away trapping the covalent protein-DNA intermediate, which can be resolved from unmodified Int on SDS-PAGE thanks to its increased size. Int variants at 40 μM and suicide CI DNA substrates (Table S6) at 20 μM were mixed in a 15 μL final reaction volume in a reaction buffer containing 25 mM HEPES pH 7.5, 100 mM NaCl, 10 mM MgCl_2_, 5% glycerol, 1 mM EDTA, 1 mM DTT and incubated for 2-4 h at 37°C. Samples were heat denatured in SDS-containing sample buffer and analyzed by electrophoresis on 12% SDS-PAGE gels. Mark12 protein size standard (Thermo Fisher) was used as marker. DNA-free protein and covalent protein-DNA complexes were detected by staining with Coomassie Brilliant Blue G250 (Sigma Aldrich). Figures show a representative of at least three independent experiments.

#### *In vitro* strand exchange assays and DNA ligation experiments

Following cleavage of a radiolabeled DNA substrate, nucleophilic attack by the 5′OH of an unlabeled partner substrate leads to strand exchange, generating a recombined product that can be detected on denaturing PAGE (see schematics in Figure S1B). Suicide DNA substrates were used to facilitate strand exchange. 2 μM 5′-^32^P-labeled CI DNA substrates (Table S6) were incubated with Int variants (at 20 μM or at different molar ratios in titration experiments) in a final reaction volume of 15 μL in a reaction buffer containing 50 mM HEPES pH 7.5, 250 mM NaCl, 5 mM DTT and 10% glycerol. Reactions were performed in the presence of a 100-fold excess of unlabeled partner DNA. After 2-4 h incubation at 37°C, the reaction was stopped by digestion with Proteinase K (Carl Roth; Proteinase K buffer 2X: 60 mM Tris-HCl pH 7.5, 20 mM EDTA, 2% SDS) at 45°C for 30 min. DNA products were precipitated with NaAc/EtOH in the presence of 24 μg/mL glycogen (Thermo Fisher). Samples were heat denaturated at 98°C for 3-5 mins in loading buffer (45% formamide, 0.5x TBE, 0.005% bromophenol blue, 0.005% xylene cyanol) and the reaction products were separated by electrophoresis on denaturing 12% polyacrylamide TBE-Urea gels. The 10/60 DNA Ladder (IDT) was used as oligonucleotide length standard. Gels were imaged with a Typhoon FLA 7000 phosphoimager (GE Healthcare). Figures show a representative of at least three independent experiments.

Ligation assays were performed following the same procedures only using half-site 5′-^32^P-labeled IR DNA substrates. These DNA oligonucleotides contain only one IR site, but are cleaved and undergo efficient ligation with another half-site substrate that mimics the cleavage product, with a free 5′OH on a complementary overhang (see schematics in Figure 4D) (Nunes-Düby et al., 1989). 2 μM 5′-^32^P-labeled half-site DNA (Table S6) was incubated with 20 μM Int and 34 μM unlabeled partner DNA in a final reaction volume of 15 μL. Ligation efficiency was compared with different partner substrates.

To determine the exact positions of cleavage, we performed activity assays with CI DNA substrates containing a phosphorothioate (PTO) modification in the DNA backbone. PTO inhibits cleavage and strand exchange by tyrosine recombinases if placed exactly at the scissile phosphate (Ennifar et al., 2003). Substrates were modified at each potential cleavage position (Table S6) and assays were performed as described above. Full-length suicide CI substrates were used to map the cleavage site at IR_L_, whereas mapping at IR_R_ required the use of half-site substrates as in the ligation assays. 6 μM 5′-^32^P-labeled PTO modified DNA substrates (Table S6) were incubated with 20 μM Int and 76 μM unlabeled DNA in a final reaction volume of 15 μL.

#### Fluorescent base flipping assays

To investigate base flipping in solution, fluorescence spectroscopy experiments were performed with DNA containing a fluorescent base analog, 2-aminopurine (2AP). 2AP is a sensitive probe of the local duplex structure, as its fluorescence is strongly quenched by base stacking in duplex DNA (Holz et al., 1998). 2AP-modified and unmodified CI5 DNA (Table S6, 500 μM) were annealed at 98°C for 2 min followed by slow cooling to 25°C. DNA at 2.15 μM were incubated with various concentrations of Int^82N^ protein (0.5-7.6 μM; stock 80 μM) in a final volume of 130 μL, at 4°C for 30-60 min in Buffer C. Fluorescence measurements were performed in 10 × 2 mm light path cuvettes (Hellma Analytics QS) at room temperature. Data was collected in a PTI QuantaMaster 8000 Fluorometer (Photon Technology International) using an excitation wavelength of 320 nm and an emission wavelength filter from 340 nm to 450 nm to reduce the influence of the natural DNA absorption and protein tryptophan fluorescence. Split aperture for excitation and emission was 5. All fluorescence emission spectra and fluorescence intensities were corrected by subtraction of control titrations with unlabeled protein-DNA complex (as described in Holz et al., 1998). Spectra are shown for a representative of at least three independent experiments. Titration experiments were repeated 6- (with Int^82N^) or 5-times (with R153A) and the mean of the corrected fluorescence intensities at different protein-DNA ratios were plotted. In some cases, a limited number of data points were excluded, due to protein precipitation in the corresponding sample. Bar graphs represent standard error of the mean (SEM) for at least three independent measurements (Figure 3; Table S3).

#### *In vivo* excision assays

*In vivo* excision assays were performed in as described in (Lambertsen et al., 2018). *E. coli* Top10 cells were transformed with two plasmids, a protein expression plasmid (pBAD-Xis/Int^FL^) and a transposon donor plasmid (DP) containing mini-Tn*1549.* Bacteria were grown in LB media with 0.2% glucose, 33 μg/mL chloramphenicol (Cm, Sigma-Aldrich) and 100 μg/mL ampicillin (Ap, Roth) to the mid-exponential growth phase; then protein expression was induced by changing the medium to 10 mL LB with 0.2% arabinose and antibiotics (as above) and diluting the culture to OD600 of 0.1. Excision of the mini-Tn*1549* transposon from the donor plasmid was tested after 3 hours of Tn*1549* Xis and Int^FL^ expression. Culture samples were spun down at 4°C at 5000 g for 5 min and supernatants were removed. Pellets were resuspended with OD600 correction (e.g., for a 0.5 mL culture sample with OD600 = 0.5 the pellet was suspended in 50 μL water), cooked and analyzed by PCR. The excised mini-Tn*1549* CI was detected with primers matching the transposon ends (CI-1 and CI-2, Table S6), and the presence or absence of the mini-Tn*1549* in the transposon donor plasmid was tested with PCR primers annealing to the transposon flanking DNA (DP-1 and DP-2, Table S6). PCR was performed using Phusion Flash High-Fidelity PCR Master Mix (Thermo Fisher) in a final volume of 25μL, following the manufacturer’s instructions. The reactions were run at 98°C for 2 min followed by 25 cycles of 98°C for 10 s, 58°C (with primers CI-1 and CI-2) or 55°C (with primers DP-1 and DP-2) for 10 s and 72°C for 30/60 s, cycles were finalised at 72°C for 2 min. Finally, the PCR products were run on a 1% agarose gel with ethidium bromide, in 1xTAE buffer (Tris-Acetate-EDTA) for 45 min to 1 hour at 100V. HyperLadder 1kb DNA ladder was used as marker.

#### HJ resolution assays

The HJ intermediate was designed to mimic the product of the first strand exchange between CI6a and a previously observed integration site (Lambertsen et al., 2018), assuming initial cleavage at IR_R_. To monitor cleavage and rejoining of each individual DNA strand in the HJ, each DNA arm was distinctly sized and differentially labeled with 5′-^32^P. Unlabeled arms were phosphorylated at the 5′ end to avoid the formation of aberrant products. The HJ was constructed by annealing four synthetic oligonucleotides (Table S6) that contain complementary crossover sequences to allow stable HJ formation. Labeled and unlabeled oligonucleotides were mixed at equal molar ratio (1 μM final concentration) in annealing buffer (10 mM Tris pH 8, 1 mM EDTA, 10 mM MgCl_2_, 100 mM NaCl), heated to 95°C for 5 min and cooled to 20°C at a rate of 1°C/min using a PCR machine. HJ formation was confirmed by electrophoresis on a 6% non-denaturing polyacrylamide gel at 100 V for 45 min. 60 μM Int^82N^ and 1 μM 5′-^32^P-labeled HJ were mixed in a final volume of 15 μL containing 50 mM HEPES pH 7.5, 250 mM NaCl, 10 mM MgCl_2_, 10% glycerol, and 1 mM DTT. Samples were incubated for 2h at 37°C and the reactions were terminated with Proteinase K digestion at 55°C for 30 min. DNA products were precipitated with NaAc/EtOH in the presence of 24 μg/mL glycogen (Thermo Fisher). Samples were heat denaturated at 98°C for 3-5 mins in loading buffer (45% formamide, 0.5x TBE, 0.005% bromophenol blue, 0.005% xylene cyanol). Reaction products were analyzed by electrophoresis on denaturing 12% PAGE TBE-Urea gels and imaged with a Typhoon FLA-7000 phosphoimager. The 10/60 DNA ladder (IDT) was used as marker. Figures show one representative of two independent experiments.

#### SAXS analysis

Small-angle X-ray scattering (SAXS) data of Int^82N^ at 1.3, 2.63, 4.8 mg/mL, and 390C variant at 0.5, 3.2, 4.18 mg/mL, were measured at beamline BM29 at the ESRF in a buffer with 50 mM HEPES pH 7.5, 750 mM NaCl, 5 mM DTT and 10% glycerol. Sample scattering curves were averaged and the buffer scattering was subtracted using the PRIMUS software (Konarev et al., 2003). Forward scattering intensity *I*(0) and radius of gyration (R_g_) were calculated with the Guinier approximation assuming that at very small angles (*s* < 1.3/R_g_) intensity is represented as *I*(*s*) = *I*(0)exp(-(sR_g_)^2^/3). The molecular weight (Mw) of the particle was estimated by protein calibration using bovine serum albumin as standard. The final Mw estimation presented is the mean value calculated from three curves at different protein concentrations.

Curves obtained at three concentrations were merged into a single curve that was used for further analysis. The maximum intramolecular distance (D_max_) and distance distribution were calculated from the scattering intensities with the GNOM software (Svergun, 1992), using the scattering angle range 0.015 < *s* < 0.50 Å^-1^. Protein flexibility was analyzed by inspection of the Kratky and the Porod-Debye plots. Kratky analysis (plotting *s*^2^·*I*(*s*) versus *s*, with scaling *s*^2^·*I*(*s*) values with the corresponding *I*(0) for each construct) showed an initial parabolic peak followed by an elevated baseline at high *s*, suggesting certain degree of flexibility in solution. Porod-Debye plots (plotting *I*·*s*^4^ versus *s*^4^) reveal an attenuated Porod–Debye plateau with associated higher particle volumes (65,200 Å^3^ for 390C and 119,000 Å^3^ for Int^82N^) and reduced protein densities (d_protein_ = 0.90 g/cm^3^ for 390C and 1.01 g/cm^3^ for Int^82N^), further confirming protein flexibility (Rambo and Tainer, 2011). Flexibility was most pronounced for the monomeric 390C construct, as reflected by both the Kratky plots and the estimated protein densities.

#### Peptide assays

Peptides (specific and scrambled; see Key Resources Table) were produced and HPLC-purified by ProteoGenix SAS, with a final purity of 97.65%. The lyophilized peptides were dissolved in degassed H_2_O by shacking at 37°C (10 min at 400 rpm) to a stock concentration of 20 mM, and diluted to a final 10 mM concentration in Buffer C. DNA binding and strand exchange assays were performed in the presence of increasing peptide concentrations (0.5-4 mM) as described above. The scrambled peptide sequence was generated using the GenScript server (https://www.genscript.com).

#### Molecular modeling

The modeling was performed following the procedure described in (Bebel et al., 2016). First, Int^82N^ tetramers were assembled by superposing individual protein domains onto λInt–COC’ post-cleavage synaptic complex structure (PDB: 1z19) by rigid structural alignment using FATCAT (Li et al., 2006). Flexible parts that could not be aligned, including the β-hairpin insertion, were isolated and modeled separately with Phyre2 (Kelley et al., 2015). As Tn*1549* Int’s helix αM is substituted with a short beta strand in the case of λInt, αM interactions were kept as observed in our structures. This means that we kept the position of the swapped αM in its binding site, while arranging the rest of the assembly according to the λInt template. This procedure is expected to provide a realistic approximation, as previous structures of other tyrosine recombinases did not reveal significant changes in the docking sites of the C terminus during different stages of the reaction (e.g., Bebel et al., 2016). Finally, the obtained tetramer was refined in HADDOCK (van Zundert et al., 2016), in order to optimize the molecular geometry.

#### Miscellaneous

Structural figures and animations were generated with Pymol (Schrödinger); sequence alignments were generated with DALI (Holm and Rosenström, 2010) and ESpript (Robert and Gouet, 2014). Protein interfaces were analyzed in PISA (Krissinel and Henrick, 2007), protein-DNA contacts in NUCPLOT (Luscombe et al., 1997) and DNA parameters in 3DNA (Lu and Olson, 2003).

### Quantification and Statistical Analysis

Reaction products from strand exchange assays with Int^82N^, R153A, and R153A-Y160A mutants were quantified using a Typhoon FLA7000 Phosphorimager and the ImageQuant TL software (GE Healthcare). DNA band intensities for substrate and product were calculated using the same box-area and the ratio between product and substrate bands was calculated for each sample (Table S4). The reported values belong to a single representative experiment.

Fluorescence intensity values are displayed as mean ± SEM of at least three independent experiments (Figure 3). Statistical analysis was performed in Excel and is shown in Table S3. Statistical significance was not tested.

### Data and Software Availability

#### Data resources

The accession numbers for the cordinates and structure factors reported in this paper are Protein Data Bank: 6EMZ (Int^82N^(R225K)-CI5 complex), 6EN1 (Int^82N^(R225K)-CI6a complex), 6EN2 (Int^82N^(R225K)-CI6b complex), 6EN0 (Int^82N^(wt)-CI5 complex), and 6EMY (Int^82N^(Y379F)-IR_R_ complex).

## Supplemental Information

Document S1. Tables S1–S6

Movie S1. Conformational Rearrangements Required for Tetramerization and Target DNA Binding, Related to Figure 7 Morphing of the dimeric Tn*1549* Int^82N^-CI DNA complex into a model of the proposed tetrameric Tn*1549* Int^82N^-CI-target DNA ternary complex (modeled based on the λInt–COC’ post-cleavage synaptic complex structure, PDB ID: 1z19). Color code as in Figure 2A. The flexible linkers between αL and αM, which could not be confidently modeled in the tetramer, are shown as dashed lines. The conformational changes can occur without clashes, involving rotation of the Int^82N^ subunits and DNA bending. In our model, the tetramer is held together by a large protein-protein interface (1393 Å^2^, calculated by PISA), comprising interactions of CB, CAT, and the C-terminal helices that are circularly swapped as in the λInt–COC’ complex. The interactions of the C-terminal helices with the CAT of the partner subunit across the tetramer interface are similar to the ones observed in our dimeric structure. In addition, complementary electrostatic interactions, unique hydrogen bonds (between K132-T161 and K132-D165) and a unique salt-bridge (K132-D165) are predicted between the CB domains. These residues are conserved in Tn*916*-like transposases, indicating their functional significance. Note that flexible interdomain linkers could not be confidently modeled, but may also participate in tetramerization.
