# Supplementary material for: Transposase-DNA Complex Structures Reveal Mechanisms for Conjugative Transposition of Antibiotic Resistance
Source: Cell. 2018 Mar 22;173(1):208–220.e20. doi: 10.1016/j.cell.2018.02.032 (PMC5871717; doi:10.1016/j.cell.2018.02.032)
Supplement: Document S1. Tables S1–S6 [file mmc1.pdf]

Cell, Volume *173*

Supplemental Information

Transposase-DNA Complex Structures Reveal Mechanisms for Conjugative Transposition of Antibiotic Resistance

Anna Rubio-Cosials, Eike C. Schulz, Lotte Lambertsen, Georgy Smyshlyaev, Carlos Rojas-Cordova, Kristoffer Forslund, Ezgi Karaca, Aleksandra Bebel, Peer Bork, and Orsolya Barabas

	Int^{82N} (225K)-CI5	Int^{82N} (225K)-CI6a	Int^{82N} (225K)-CI6b	Int^{82N} (WT)-CI5	Int^{82N}(379F)-IR_R	Int^{82N}(379F)-IR_R Se-MET
**Data Collection**						
X-ray source	EMBL PETRAIII P13	EMBL PETRAIII P13	EMBL PETRAIII P13	EMBL PETRAIII P13	EMBL PETRAIII P13	EMBL PETRAIII P13
Space group	$P2_1$	$P2_1$	C2	$P2_1$	$P2_1$	$P2_1$
a, b, c (Å)	64.59, 125.06, 77.98	77.74, 123.89, 77.76	79.93, 132.73, 125.03	77.66, 124.32, 77.40	77.23, 121.68, 77.23	77.7, 122.6, 77.9
α, β, γ (°)	90.00, 96.53, 90.00	90.00, 118.21, 90.00	90.00, 90.10, 90.00	90.00, 117.69, 90.00	90.00, 118.12, 90.00	90.0, 117.4, 90.0
Wavelength (Å)	0.9537	0.9763	0.9763	0.9763	0.9773	0.9773
Resolution	48.36 - 2.52 (2.67 - 2.52)[1]	68.47 - 2.60 (2.67 - 2.60)	68.47 - 2.48 (2.55 - 2.48)	46.04 - 2.80 (2.87 - 2.80)	45.38 - 2.50 (2.64 - 2.50)	50.0 – 2.80 (2.87 - 2.80)
R_{sym} (%)	9.6 (94.1)	13.3 (130.9)	6.0 (92.9)	10.2 (141.5)	6.5 (83.5)	10.9 (98.8)
R_{meas} (%)	10.6 (107.1)	15.6 (152.3)	7.4 (115.6)	12.5 (173.9)	7.6 (97.4)	12.9 (117.1)
I/σ (*I*)	13.29 (1.27)	8.19 (1.02)	11.85 (1.00)	10.67 (0.95)	11.7 (1.60)	8.79 (1.35)
Completeness (%)	96.6 (79.3)	99.3 (97.2)	92.5 (87.0)	96.6 (98.5)	96.5 (98.0)	97.1 (98.2)
Redundancy	5.6 (7.3)	3.8 (3.8)	2.6 (2.4)	2.9 (2.9)	3.8 (3.8)	3.5 (3.5)
Number of observations	222286 (20680)	152339 (10875)	112309 (7139)	88721 (6775)	158579 (23479)	213781 (16250)
CC (1/2) (%)	99.8 (55.2)	99.4 (49.2)	99.8 (44.4)	99.5 (62.2)	99.9 (65.9)	99.4 (54.7)
**Refinement**						
Resolution (Å)	48.36-2.79	68.47 – 2.67	68.47 – 2.67	46.04 – 2.80	38.60[2] – 2.50	
Number of reflections total/ free set	29672/1467	36788/1868	34209/1631	31128/1625	79794/ 4185	
R_{work}/R_{free} (%)	19.7/25.6	18.8/24.4	18.5/24.4	22.7/25.4	16.0/18.4	
Rmsd bond lengths (Å) / angles (°)	0.002/0.399	0.004/0.594	0.003/0.500	0.003/0.579	0.005/0.767	
Average B-value (Å^2)	67.0	65.0	75.0	95.0	71.0	
Ramachandran favored (%)	97	98	98	96	96	
Ramachandran outliers (%)	0	0	0	0	0	

[1] Numbers in parentheses show the statistic for the highest resolution shell.
[2] The low resolution cut-off was set to be consistent with the FreeR set inherited from other datasets.

Table S1. Summary of crystallographic data and refinement statistics, Related to Figure 2

step	Shift	Slide	Rise	Tilt	Roll	Twist
AT/AT	0.12	-0.27	2.96	1.53	-7.41	38.19
TA/TA	0.42	0.11	3.44	5.44	-8.71	39.61
AA/TT	0.25	-0.32	3.36	0.91	-4.34	42.86
AC/GT	0.72	-0.55	3	1.27	5.8	30.3
CC/GG	-0.46	-0.68	3.57	-3.45	7.48	30.88
CT/AG	-0.01	-0.18	3.08	2.54	9.07	32.39
TA/TA	0.01	0.75	3.27	-2.39	1.3	36.99
AA/TT	0.29	-0.48	3.45	0.87	0.21	38.79
AA/TT	0.03	-0.74	3.09	0.78	-3.61	32.92
AA/TT	0.28	-0.87	3.25	-0.01	-6.33	34.67
AT/AT	-0.61	-1.43	3.07	-5.26	2.99	34.75
TT/AA	0.16	-0.64	4.16	5.48	-20.69	31.6
TT/AA	-0.5	-0.29	3.1	-0.69	-0.57	39.01
TT/AA	1.2	0.16	3.39	1.56	2.78	37.73
TA/TA	----	----	----	----	----	----
AG/CT	----	----	----	----	----	----
GA/TC	----	----	----	----	----	----
AA/TT	-0.96	-0.08	3.7	1.12	-2.1	37.31
AA/TT	0.62	-0.42	3.13	-0.21	-0.79	38.74
AA/TT	-0.11	-0.5	3.8	-3.33	-18.97	32.07
AT/AT	-0.09	-1.28	3.21	4.63	1.67	33.8
TT/AA	0.26	-0.92	3.21	-2.05	-5.86	33.95
TA/TA	-0.59	-0.61	3.43	-2.87	0.09	37.08
AT/AT	-0.71	-0.85	3.43	-3.06	-3.05	36.08
TA/TA	0.09	0.38	3.14	-0.26	1.32	34.84
AT/AT	-0.11	-0.73	3.09	-1.8	6.3	33.08
TG/CA	0.26	-0.33	2.82	0.98	10.51	27.83
GG/CC	-0.74	-0.14	3.07	-1.22	-1.17	34.15
GG/CC	0.27	-1	3.4	1.01	6.83	35.94
GA/TC	0.09	-0.45	3.59	-1.25	-19.29	38.81
average	0.07	-0.31	3.43	0	-1.1	36.23
SD	0.56	0.75	0.63	2.88	8.2	6.36

Table S2. DNA conformational parameters in the Int^{82N}-CI5 structure as calculated by 3DNA, Related to Figures 2 and 3

Listed are the six rigid body parameters that describe the position and orientation of one base pair relative to another (shift, slide, rise, tilt, roll and twist) for each dimer step in the CI5 sequence. Values are missing at the crossover region, where regular base pairing is disrupted due to base flipping, preventing confident determination of the rigid body parameters. SD refers to standard deviation. http://x3dna.org/

	Int^{82N} - CI5-F		R153A - CI5-F		
Protein: DNA ratio	I(mean)	SEM	I(mean)	SEM	I(wt) / I(R153)
0.42	2382	3617	-4979	4791	0
0.84	24317	5396	3442	3021	7
1.2	32680	7701	15551	9196	2
1.68	63845	8480	36287	14444	2
2	64862	7411	14350	4121	5
2.5	96497	11428	42484	13392	2

Table S3. Fluorescence intensity values for Int^{82N} and R153A mutant in complex with a 2AP-modified CI5 DNA (CI5-F) at different protein-DNA ratios, Related to Figure 3

I(mean) refers to the mean intensity and SEM shows the standard error of the mean for 3-6 independent experiments.

sample	concentration (μM)	I[product]	I[substrate]	I[product]/I[substrate]
Int^{82N}	2.8	1316	884	1.49
Int^{82N}	5.6	1783	920	2.04
Int^{82N}	16.8	3406	984	3.7
R153A	2.8	1290	1178	1.31
R153A	5.6	1063	1417	0.9
R153A	16.8	1044	2016	0.74
R153A-Y160A	2.8	1050	1074	0.52
R153A-Y160A	5.6	1026	1941	0.95
R153A-Y160A	16.8	1231	1496	0.63

Table S4. Quantification of strand exchange products with Int^{82N}, R153A, and R153A-Y160A mutants, Related to Figure 3

The intensities (I) of substrate and product bands were quantified on denaturing PAGE gels using ImageQuantTL (GE Healthcare). The boxes used for quantification had the same area in all cases.

Hydrophobic contacts				Hydrophilic contacts			
residue	buried area (%)	solvation energy (Δ^iG, kcal/M)	identity (%)	residue	buried area (%)	solvation energy (Δ^iG, kcal/M)	identity (%)
**Core**				**Core**			
Gly221	40	-0.08	19.6	Asn241	30	-0.14	79.6
Arg242	10	0	53.2	Gly358	40	-0.17	59.2
Val243	80	0.34	38.8	Asn360	40	-0.11	90.4
Lys271	40	0.37	8.8	Lys362	70	0	73.2
Ile272	90	0.03	24.4	**αM**			
Pro273	90	0.97	96.4	Tyr380	80	0.44	93.6
Asn275	20	0.15	2.4	Glu391	70	0.16	90.4
Phe350	50	0.17	75.6	Arg394	20	-0.26	87.6
Leu354	60	0.53	45.2				
Ala357	40	0.14	38.8				
Met359	100	0.86	92.8				
Pro361	100	0.71	93.6				
Ala363	100	0.61	37.6				
Tyr366	40	0.69	94.0				
Ile367	100	0.37	74.8				
Ile373	60	0.53	92.8				
Thr374	40	0.06	37.2				
Leu377	100	1	63.2				
Asn378	10	0.06	66.8				
**αM**							
Ala381	20	-0.17	90.8				
His382	10	0.12	93.6				
Ala383	80	0.62	65.6				
Thr384	10	0.08	50				
Phe385	90	1.69	49.2				
Ser387	10	0.03	54.8				
Ala388	90	0.75	85.2				
Arg389	50	0.09	6.8				
Met392	100	2.93	38.8				
Glu393	10	0.04	33.6				
Leu395	50	1.16	89.2				
Ala396	20	0.34	25.6				

Table S5. List of interface residues in the Int^{82N} dimer interface based on PISA (Krissinel and Henrick, 2007), Related to Figures 6 and S7

Hydrophobic or hydrophilic contacts were classified depending on Δ^iG, the solvation energy of the corresponding residue. Positive Δ^iG for a residue has a negative contribution to the overall solvation energy gain, indicating hydrophobic contact. Buried area indicates the solvent-accessible surface area of the corresponding residue that becomes buried upon forming the interface. The buried area is shown as a percentage of the total solvent-accessible surface area for each residue. The identity (%) column indicates the percentage of sequences having the same residue in the corresponding position across Tn*916*-like transposases. Residues listed under the heading "Core" are located in the core of Int's CAT domain and residues shown under "αM" are in the C-terminal helix of the protein.

	**PCR primers**	Modification
R225K	CACAGTTCGCTAATCTTCAGACCGGTGCCCA	5' Phos
IntΔβ	CCGCGTGATTATTGTTAGCGGTGGTAAAACCCAGAGCGGTGTTCG	5' Phos
R153A	CATCAACAATGATAAAGCTAGCCTGAAAGCAGC	5' Phos
Y160A	GTAGCCTGAAAGCAGCATTTGCTACCGCAATTCAGGATGATTGC	5' Phos
Y379F	GCAATATTACCATGACCCTGAACTTTTATGCCCATGCAACCTTTGATAGCGCACG	5' Phos
Y380F	GCAATATTACCATGACCCTGAACTATTTTGCCCATGCAACCTTTGATAGCGCACG	5' Phos
2YF	GCAATATTACCATGACCCTGAACTTTTTTGCCCATGCAACCTTTGATAGCGCACG	5' Phos
381C	CATGACCCTGAACTATTATGCCTAACACCACCACCACCACTG	5' Phos
384C	GAACTATTATGCCCATGCAACCTAACACCACCACCACCACTG	5' Phos
390C	CCTTTGATAGCGCACGTGCATAACACCACCACCACCACTG	5' Phos
R225K-Int^{FL}	GGGCTTaaaATTTCGGAACTGTGCGGACTG	5' Phos
R153A-Int^{FL}	GACCATCAATAACGACAAGgcgTCCCTGAAAGCGGC	5' Phos
Y160A-Int^{FL}	GCGGCTTTTgcgACCGCCATACAGGACGATTGC	5' Phos
CI-1	GCGGGATCCTGTTCTCCCAT	
CI-2	ACGCAAGCTTCGATTCCGCAAG	
DP-1	GAGAGCAGCTGAAGTTACCC	
DP-2	GTAACTTTAAACGGACCACTAGGAG	
	**Crystallization oligonucleotides**[1]	Purification
CI5	TGCGATAACCTAAAATTTTatagcAAAATTATATGGGATTTTAG	PAGE
CI5’	CTAAAATCCCATATAATTTTgctatAAAATTTTAGGTTATCGCT	PAGE
CI6a	TGCGATAACCTAAAATTTTattttcAAAATTATATGGGATTTTAG	PAGE
CI6a’	CTAAAATCCCATATAATTTTgaaaatAAAATTTTAGGTTATCGCT	PAGE
CI6b	TGCGATAACCTAAAATTTTccctttAAAATTATATGGGATTTTAG	PAGE
CI6b’	CTAAAATCCCATATAATTTTaaagggAAAATTTTAGGTTATCGCT	PAGE
IR_R	attttcAAAATTATGGGATTTTAG	PAGE
IR_R’	CTAAAATCCCATATAATTTT	PAGE
	**DNA oligonucleotides for cleavage and strand exchange assays**[2]	Modification
CI5_full	CTAAAATCCCATATAATTTTgctatAAAATTTTAGGTTATCGCT	5' Phos
CI5_nicked1	TGCGATAACCTAAAATTTTa	
CI5_nicked2	tagcAAAATTATATGGGATTTTAG	5' Phos
CI6a_full	CTAAAATCCCATATAATTTTgaaaatAAAATTTTAGGTTATCGCTG	5' Phos
CI6a_nicked1	CAGCGATAACCTAAAATTTTa	
CI6a_nicked2	ttttcAAAATTATATGGGATTTTAG	5' Phos
CI6b_full	CTAAAATCCCATATAATTTTaaagggAAAATTTTAGGTTATCGCTG	5' Phos
CI6b_nicked1	CAGCGATAACCTAAAATTTTc	
CI6b_nicked2	cctttAAAATTATATGGGATTTTAG	5' Phos
	**DNA oligonucleotides with 2-aminopurine (2AP) modification**[1,3]	Modification
CI5-F	TGCGATAACCTAAAATTTTtt ag/2AP/AAAATTATATGGGATTTTAG	i2AmPr
CI5-F’	CTAAAATCCCATATAATTTTccta/2AP/AAAATTTTAGGTTATCGCT	i2AmPr
CI6b-F	CAGCGATAACCTAAAATTTTccctt/2AP/AAAATTATATGGGATTTTAG	i2AmPr

CI6b-F’	CTAAAATCCCATATAATTTTtaagg/2AP/AAAATTTTAGGTTATCGCTG	i2AmPr
CI5-IR	TGCGATAACCTAAAATTTTatagc/2AP/AAATTATATGGGATTTTAG	i2AmPr
CI5-IR’	CTAAAATCCCATATAATTTTgctat/2AP/AAATTTTAGGTTATCGCT	i2AmPr
CI5-Co	TGCGATAACCTAAAATTTTata/2AP/cAAAATTATATGGGATTTTAG	i2AmPr
CI5-Co’	CTAAAATCCCATATAATTTTgct/2AP/tAAAATTTTAGGTTATCGCT	i2AmPr
	**DNA oligonucleotides with phosphorothioate (PTO) modification**[4]	Modification
CI5_PTO_0’	CTAAAATCCCATATAATTTT*gcta	PTO
CI5_PTO_-1’	CTAAAATCCCATATAATTT*Tgcta	PTO
CI5_PTO_-1’,0’	CTAAAATCCCATATAATTT*T*gcta	PTO
CI5_PTO_0	TGCGATAACCTAAAATTTT*at a	PTO
CI5_PTO_-1	TGCGATAACCTAAAATTT*Tata	PTO
CI5_PTO_-1,0	TGCGATAACCTAAAATTT*T*ata	PTO
CI6a_PTO_0’	CTAAAATCCCATATAATTTT*gaaa	PTO
CI6a_PTO_-1’	CTAAAATCCCATATAATTT*Tgaaa	PTO
CI6a_PTO_-1’,0’	CTAAAATCCCATATAATTT*T*gaaa	PTO
CI6a_PTO_0	TGCGATAACCTAAAATTTT*att	PTO
CI6a_PTO_-1	TGCGATAACCTAAAATTT*Tatt	PTO
CI6a_PTO_-1,0	TGCGATAACCTAAAATTT*T*att	PTO
	**Half-site oligonucleotides**[1,5]	Modification
IR$_{L}$	AGCGATAACCTAAAATTTTatagc	
IR$_{L}$’_T-1’	TgctatAAAATTTTAGGTTATCGCT	
IR$_{L}$’_*g*0’	gctatAAAATTTTAGGTTATCGCT	
IR$_{L}$’_T-1’^{P}	TgctatAAAATTTTAGGTTATCGCT	5' Phos
IR$_{R}$’	CTAAAATCCCATATAATTTTgctat	
IR$_{R}$_T-1	TatagcAAAATTATATGGGATT	
IR$_{R}$_*a*0	atagcAAAATTATATGGGATT	
IR$_{R}$_*t*1	tagcAAAATTATATGGGATT	
IR$_{R}$_T-1^{P}	TatagcAAAATTATATGGGATT	5' Phos
IR$_{L}$’_T	TgctatAAAATTTTAGGTTATCGCT	
IR$_{L}$’_A	AgctatAAAATTTTAGGTTATCGCT	
IR$_{L}$’_C	CgctatAAAATTTTAGGTTATCGCT	
IR$_{L}$’_G	GgctatAAAATTTTAGGTTATCGCT	
IR$_{L}$’_T^{P}	TgctatAAAATTTTAGGTTATCGCT	5' Phos
IR$_{R}$_T	TatagcAAAATTATATGGGATT	
IR$_{R}$_A	AatagcAAAATTATATGGGATT	
IR$_{R}$_C	CatagcAAAATTATATGGGATT	
IR$_{R}$_G	GatagcAAAATTATATGGGATT	
IR$_{R}$_T^{P}	TatagcAAAATTATATGGGATT	5' Phos
IR$_{L}$_6a	AGCGATAACCTAAAATTTTattttc	
IR$_{L}$’_6a_T	TgaaaatAAAATTTTAGGTTATCGCT	
IR$_{L}$’_6a_A	AgaaaatAAAATTTTAGGTTATCGCT	
IR$_{L}$’_6a_C	CgaaaatAAAATTTTAGGTTATCGCT	
IR$_{L}$’_6a_G	GgaaaatAAAATTTTAGGTTATCGCT	
IR$_{L}$’_6a_T^{P}	TgaaaatAAAATTTTAGGTTATCGCT	5' Phos
IR$_{R}$’_6a	CTAAAATCCCATATAATTTTgaaaat	

IR_R_6a_T	TattttcAAAATTATATGGGATT	
IR_R_6a_A	AattttcAAAATTATATGGGATT	
IR_R_6a_C	CattttcAAAATTATATGGGATT	
IR_R_6a_G	GattttcAAAATTATATGGGATT	
IR_R_6a_T^P	TattttcAAAATTATATGGGATT	5' Phos
	**DNA oligonucleotides for crossover competition assays**	Size (nt)
Cross5	TGCTA	5
Cross5_[5'P]	[Phos]TGCTA	5
Cross10	TGCTATAAAA	10
	**DNA oligonucleotides for Holliday Junction binding assays**[6]	Size (nt)
T_L_EMSA (T_L-IR_L)	AGTGAACAGCCCACAAAATTTTGAAAATAAAATTTTAGGTTATCGC TGGCTAGTCCATGC	60
IR_L*_EMSA (IR_L-IR_R)	GCATGGACTAGCCAGCGATAACCTAAAATTTTATTAATAAAATTA TATGGGATTT	55
T_R*_EMSA (T_R-T_L)	CCAAATTTTTTACCGGGTTTTGAATTCAAAATTTTGTGGGCTGTTCA CT	49
IR_R_EMSA (IR_R-T_R)	AAATCCCATATAATTTTATTTTCAAAACCCGGTAAAAAATTTGG	44
	**DNA oligonucleotides for Holliday Junction resolution assays**[6]	Size (nt)
T_L* (T_L-IR_L)	ACAGCCCACAAAATTTTGAAAATAAAATTTTAGGTTATCGCTGG	44
IR_L* (IR_L-IR_R)	CCAGCGATAACCTAAAATTTTATTAATAAAATTATATGGGATTTTA GAT	49
T_R* (T_R-T_L)	AAATTTTTTACCGGGTTTTGAATTCAAAATTTTGTGGGCTGT	42
IR_R* (IR_R-T_R)	ATCTAAAATCCCATATAATTTTATTTTCAAAACCCGGTAAAAAATT T	47
Product marker 44	TGTC GGGTGTTTTAAAACTTTTATTTTAAAATCCAATAGCGACC	44
Product marker 49	GGTCGCTATTGGATTTTAAAATAATTATTTTAATATACCCTAAAATC TA	49
Product marker 42	TTTAAAAAATGGCCCAAAACTTAAGTTTTAAAACACCCGACA	42
Product marker 47	TAGATTTTAGGGTATATTAAAATAAAAGTTTTGGGCCATTTTTTAA A	47

[1] The oligonucleotides labelled with ' represent the 'strand as in Figures 2A and S3E.

[2] CI5_nicked1 and CI5_nicked2 were annealed with CI5_full, to generate a double stranded suicide DNA substrate with a nick positioned 2 nts downstream of the cleavage site. Similarly, CI6a_nicked1 and CI6a_nicked2 were annealed with CI6a_full to generate the CI6a suicide DNA, and CI6b_nicked1 and CI6b_nicked2 were annealed with CI6b_full to generate the CI6b suicide DNA.

[3] The DNA oligonucleotides used for fluorescent assays contain 2-aminopurine (2AP) modification at various positions. F: 2AP at the flipped-out base on both strands, IR: 2AP adjacent to the flipped-out base inside the IR, Co: 2AP next to the flipped-out base in the crossover region.

[4] PTO modification is indicated with an asterisk in the DNA sequence.

[5] Half-site oligonucleotides labelled IR_R and IR_L represent the CI5 sequence; oligonucleotides labelled with 6a contain the CI6a sequence.

[6] The synthetic Holliday Junction intermediate was created by annealing the four ssDNA oligonucleotides listed.

Table S6. List of DNA oligonucleotides used for cloning, crystallization, DNA cleavage, strand exchange, ligation and fluorescent assays, Related to STAR Methods
